# Topographic confinement of epithelial clusters induces epithelial-to-mesenchymal transition in compliant matrices

**DOI:** 10.1038/srep18831

**Published:** 2016-01-05

**Authors:** Samila Nasrollahi, Amit Pathak

**Affiliations:** 1 Department of Mechanical Engineering and Materials Science, Washington University, Saint Louis, MO 63130, USA

## Abstract

Epithelial cells disengage from their clusters and become motile by undergoing epithelial-to-mesenchymal transition (EMT), an essential process for both embryonic development and tumor metastasis. Growing evidence suggests that high extracellular matrix (ECM) stiffness induces EMT. In reality, epithelial clusters reside in a heterogeneous microenvironment whose mechanical properties vary not only in terms of stiffness, but also topography, dimensionality, and confinement. Yet, very little is known about how various geometrical parameters of the ECM might influence EMT. Here, we adapt a hydrogel-microchannels based matrix platform to culture mammary epithelial cell clusters in ECMs of tunable stiffness and confinement. We report a previously unidentified role of ECM confinement in EMT induction. Surprisingly, confinement induces EMT even in the cell clusters surrounded by a soft matrix, which otherwise protects against EMT in unconfined environments. Further, we demonstrate that stiffness-induced and confinement-induced EMT work through cell-matrix adhesions and cytoskeletal polarization, respectively. These findings highlight that both the structure and the stiffness of the ECM can independently regulate EMT, which brings a fresh perspective to the existing paradigm of matrix stiffness-dependent dissemination and invasion of tumor cells.

Epithelial-to-mesenchymal transition (EMT) enables epithelial cells to escape their native colony, attain motile phenotypes, and migrate through complex tissue barriers. While a coordinated form of EMT is essential during embryonic development, its dysfunctional versions can cause tissue fibrosis and cancer progression^1^,^2^. The clusters and colonies of epithelial cells reside in complex microenvironments of varying mechanical properties, such as stiffness, topography, porosity, and dimensionality. There is growing evidence that mechanical cues present in the extracellular matrix (ECM), including stiffness and topography, regulate mechanosensitive subcellular pathways and influence cellular functions such as motility, differentiation, and fate decisions^3^,^4^,^5^,^6^,^7^,^8^,^9^. In particular, cells on stiffer ECMs generate higher actomyosin forces and form stronger adhesions, both of which are known to weaken cell-cell junctions^10^,^11^. Several recent studies have demonstrated a direct relationship between ECM stiffness and the induction of EMT in various cell types and matrices. On stiff collagen-based matrices, mammary epithelial cells undergo EMT and attain an invasive phenotype^10^,^12^,^13^. On stiff fibronectin substrates, alveolar epithelial cells become increasingly contractile and activate transforming growth factor-β (TGF–β), which leads to fibrosis-associated EMT^14^,^15^. Thus, it is likely that EMT might be sensitive to various mechanical properties that define the surrounding ECM. While the field has made great strides in understanding stiffness-dependent EMT^12^,^15^,^16^,^17^, the effects of topographical properties of the ECM on EMT remain largely unknown. This represents an important gap in knowledge given that tissue environments often vary in topography and confinement without significant variation in stiffness. For example, at the onset of carcinoma invasion, cell clusters being released from a tumor encounter discontinuities in the surrounding tissue, which results in varying degrees of ECM confinement around these epithelial clusters of tumor cells^18^,^19^. Given the mechanical complexity of microenvironments surrounding the tumor cell clusters, it is of critical importance to understand how ECM topography combines with stiffness to induce EMT.

In this work, we ask whether ECM stiffness and confinement can independently induce EMT. We also assess how subcellular mechanisms of force generation and adhesion formation influence this ECM-dependent mesenchymal transition. To conduct these studies, we employ a device that allows the culture of epithelial colonies in polyacrylamide (PA) channels of tunable stiffness and confinement. We cultured MCF10A mammary epithelial cells in this matrix platform and observed greater EMT inside narrower channels. Surprisingly, increased confinement leads to enhanced mesenchymal transition even in soft ECMs, which otherwise maintain an epithelial phenotype in unconfined (wide) channels. In narrow channels, cells adopt an elongated morphology, which might be indicative of their ability to sense ECM confinement by rearranging their cytoskeleton. Indeed, after the disruption of the cytoskeletal structure by pharmacological inhibition of microtubules and nonmuscle myosin II, cells lost their ability to undergo EMT in a confinement-dependent manner. We also found that inhibition of focal adhesion kinase (FAK) disabled the effect of ECM stiffness on EMT. Our results suggest two different cellular mechanisms for confinement- and stiffness-sensitive EMT. First, the ability of cells to generate active actomyosin forces, maintain cytoskeletal structure through microtubules, and attain elongated shapes might play a central role in enabling confinement-sensitive EMT. Second, cell-ECM adhesions might be key mediators for triggering a mechano-transductive signaling cascade that weakens cell-cell adhesions and induces EMT. Taken together, these results bring a fresh perspective to the existing paradigm of matrix stiffness-dependent EMT and highlight that ECM confinement alone can disrupt the integrity of epithelial entities.

## Results

### Fabrication of a matrix platform with topographic confinement around epithelial clusters

**Figure 1:**
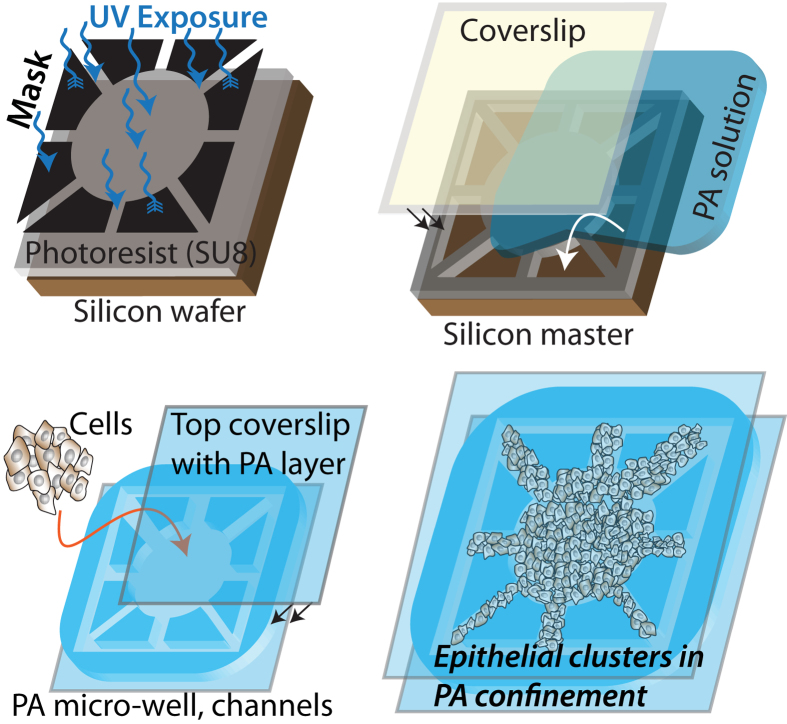
Fabrication of microchannel-based culture platform for epithelial clusters. Schematic describing the fabrication steps for molding polyacrylamide (PA) hydrogels of prescribed stiffness against photolithographically fabricated masters of defined topography. The device is designed such that an epithelial colony of MCF10A cells can be seeded in the center well and allowed to grow into peripheral PA-channels of defined stiffness and confinement.

To mimic the growth of epithelial clusters in microenvironments of varying stiffness and topography, we fabricated an *in vitro* matrix platform as a polyacrylamide microchannels-based scaffold^4^,^20^. In this system, we used photolithography techniques to fabricate silicone masters of defined topography, as shown in Fig. 1, where channel width is prescribed in the mask design and the depth is dictated by the thickness of the photoresist layer. We polymerized PA solutions of specified monomer-crosslinker ratios against these masters, which yielded PA substrates of defined stiffness and topography. We estimated average channel height to be approximately 16 μm, measured by mixing fluorescent beads in the PA solution and performing confocal microscopy on polymerized PA gels (Supplementary Fig. 1). Our substrate design allows for the culture of an epithelial colony in a center chamber of 5 mm diameter surrounded by channels of widths ranging between 20–200 μm. After seeding the colony, we sealed the device by a PA layer of matching stiffness on top. In this system, as the epithelial colony grows beyond the center chamber, its branches are constrained within the channels that surround the chamber. Thus, we were able to create a defined level of topographic confinement around epithelial clusters with independently tunable ECM stiffness. We chose PA composition, as calibrated previously^4^,^20^,^21^, corresponding to elastic moduli 1, 20, or 120 kPa.

### Enhanced EMT signatures in stiffer and narrower channels

**Figure 2:**
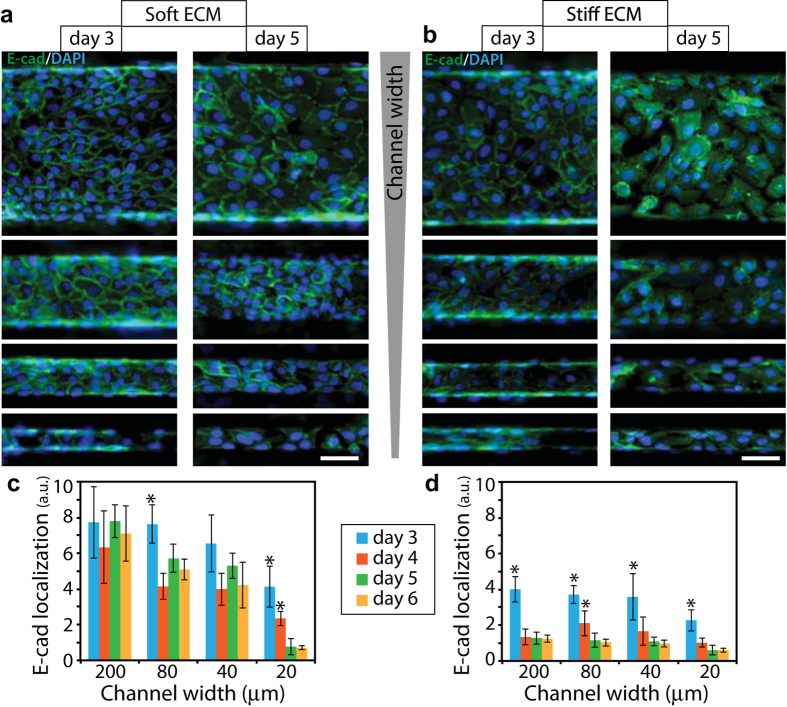
Change in E-cad expression over time in ECMs of varying stiffness and confinement. Representative immunofluorescence images of E-cad (green) expression with DAPI (blue) in MCF10A cell clusters cultured for either 3 or 5 days inside channels made of **(a)** soft, 1 kPa, and **(b)** stiff, 120 kPa, PA gels, with channel widths ranging between 20–200 μm; see split DAPI and E-cad images in Supplementary Figures 3–5. Scale bar = 50 μm. Average fluorescence intensity of membrane localized E-cad in epithelial clusters seeded for 3, 4, 5, or 6 days, inside **(c)** soft and **(d)** stiff PA channels of varying widths. ^*^p < 0.05 with respect to day 5 values for any given channel condition. N > 7 clusters of numerous cells, from at least two separate experiments (see details in Methods).

To better understand how ECM confinement and stiffness might influence EMT in the context of breast cancer, where malignancies commonly develop from epithelial origin^22^,^23^, we cultured mammary epithelial MCF10A cells in our PA-channels platform. We examined the progression of EMT in epithelial clusters confined inside channels of defined properties by performing confocal microscopy and imaging the distribution of E-cadherin (E-cad), an epithelial marker^1^,^24^, after days 3, 4, 5 and 6 of cell seeding (Fig. 2). From these images, we quantified E-cad localization near the cell membrane (see Methods and Supplementary Figure 2) because this molecule delocalizes from the membrane upon weakening of cell-cell junctions and induction of EMT. In wide channels made of soft (1 kPa) ECM, E-cad localization levels were the highest and the differences among days 3–6 were negligible. Thus, soft unconfined ECMs protect against EMT, consistent with previous studies^12^,^15^,^16^,^17^, regardless of culture duration. Inside narrower channels made of soft ECM, the E-cad localization on day 3 was generally higher than that on days 5 or 6, which indicates a progression of EMT over time in confined environments even with low ECM stiffness. On stiff (120 kPa) ECM, E-cad localization on day 5 was consistently lower than that on day 3, regardless of channel width. Since the E-cad localization and the associated EMT induction appears to reach a steady state at day 5, we decided to analyze EMT markers only at day 5 for all studies presented henceforth. To examine whether this mechano-sensitive induction of EMT holds true for another epithelial cell line, we evaluated E-cad localization in Madin-Darby Canine Kidney (MDCK) cells cultured inside channels of varying stiffness and confinement (see Supplementary Figure 6). Here, MDCK cells underwent confinement-sensitive EMT, similar to the trend observed for MCF10A cells. However, MDCK cells did not undergo stiffness-sensitive EMT, which is consistent with a previous study^17^. Thus, mechano-sensitive induction of EMT is not cell-line independent. In this manuscript, we focus on studying the regulation of confinement- and stiffness-sensitive EMT in MCF10A cells.

**Figure 3:**
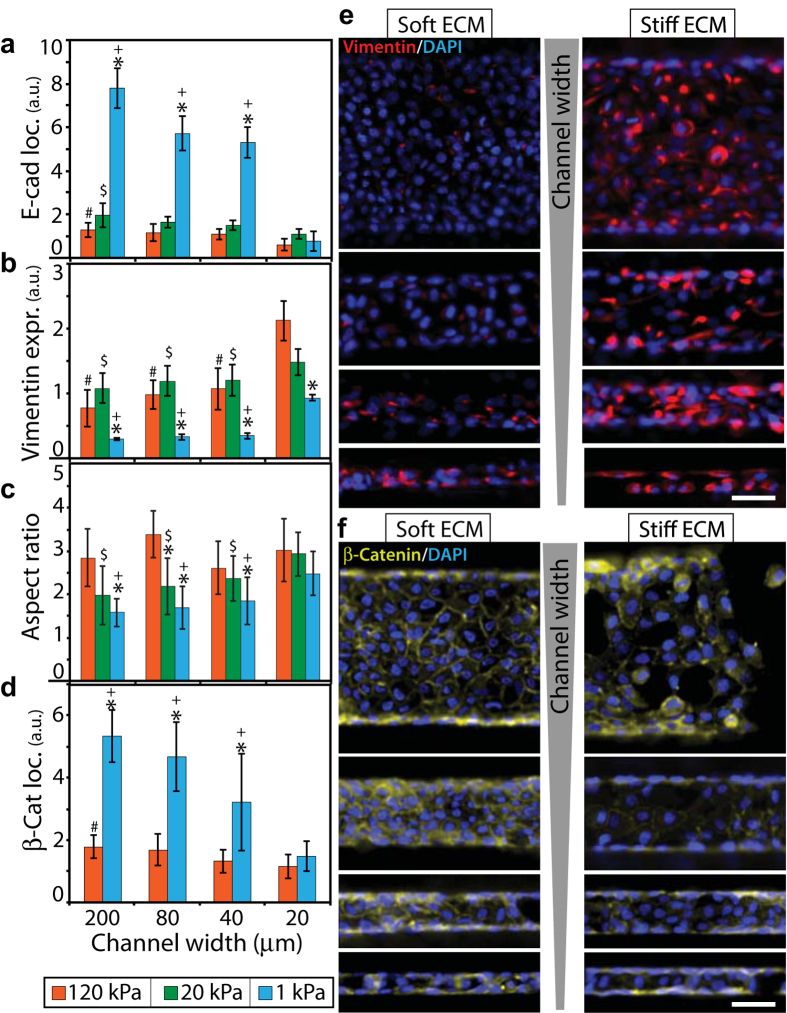
Regulation of EMT by ECM confinement and stiffness. **(a)** Average fluorescence intensity of membrane localized E-cad, **(b)** average fluorescence intensity of vimentin expression, **(c)** Elongation of individual cells measured as aspect ratio, and **(d)** average fluorescence intensity of membrane localized β-catenin in MCF10A epithelial clusters after 5 days of culture inside PA channels of varying widths and stiffness between 1–120 kPa. Representative immunofluorescence images of **(e)** vimentin (red) and **(f)** β-catenin (yellow) expressions with DAPI (blue) in MCF10A cell clusters cultured inside channels made of soft (1 kPa) and stiff (120 kPa) PA gels after day 5, with channel widths ranging between 20–200 μm; see split-channel images in Supplementary Figures 7,8. Scale bar = 50 μm. ^*^p < 0.05 with respect to stiff ECM. ^+^*p* < 0.05 with respect to narrow (20 μm) channels for 1 kPa stiffness. ^$^*p* < 0.05 with respect to narrow channels for 20 kPa stiffness. ^#^*p* < 0.05 with respect to narrow channels for 120 kPa stiffness. N > 8 clusters of numerous cells, from at least three separate experiments (see details in Methods).

At steady state, we found that E-cad membrane localization of MCF10A cells inside soft (1 kPa) channels decreased with channel width, which indicated a rise in EMT in more confined ECMs (Figs 2 and 3a). To confirm these findings with a mesenchymal marker, we quantified vimentin distribution for cell clusters in all channel widths. We found that vimentin expression increased with decreasing channel width (Fig. 3b,e), which corresponds to enhanced EMT in narrower channels. We also confirmed these findings by quantifying junctional localization of β-catenin (β-cat), an additional EMT marker, which is a key component of cell-cell junctions and is expected to diffuse into the cytoplasm upon EMT induction (Fig. 3d,f). Taken together, E-cad, β-cat and vimentin comparisons across channel widths in soft ECMs presented strong evidence for confinement-dependent EMT. Next, to examine the effect of ECM stiffness on EMT regulation in confinement, we repeated these experiments in channels made of 20 and 120 kPa PA gels. In these stiffer channels, we found that membrane localization of E-cad and β-cat decreased, while vimentin expression increased, for higher ECM stiffness in all channel widths (Figs 2 and 3a–d), all of which correspond to enhanced EMT in stiffer ECMs. Our results revealed that stiffness-dependent EMT previously observed on two-dimensional matrices^10^,^15^,^16^,^17^ also holds true in confined ECM settings. While the effect of confinement on EMT induction was more pronounced in soft (1 kPa) ECMs, it persisted in stiffer (20 and 120 kPa) ECMs. Given this direct dependence of EMT markers on channel width, we hypothesized that topographic confinement of epithelial clusters might force the cells to attain elongated morphology and weaken cell-cell junctions. To pursue this hypothesis, we first quantified elongation of individual cells in epithelial clusters inside channels in terms of aspect ratio. As shown in Fig. 3c, we found that cells in narrower channels were more elongated for 1 and 20 kPa stiffness conditions. However, cells were maximally elongated in channels of 120 kPa stiffness regardless of channel width. Thus, the elongation of epithelial cells in narrow channels correlated with confinement-dependent induction of EMT.

### Disruption of cytoskeletal structure blunts confinement-sensitive EMT

**Figure 4:**
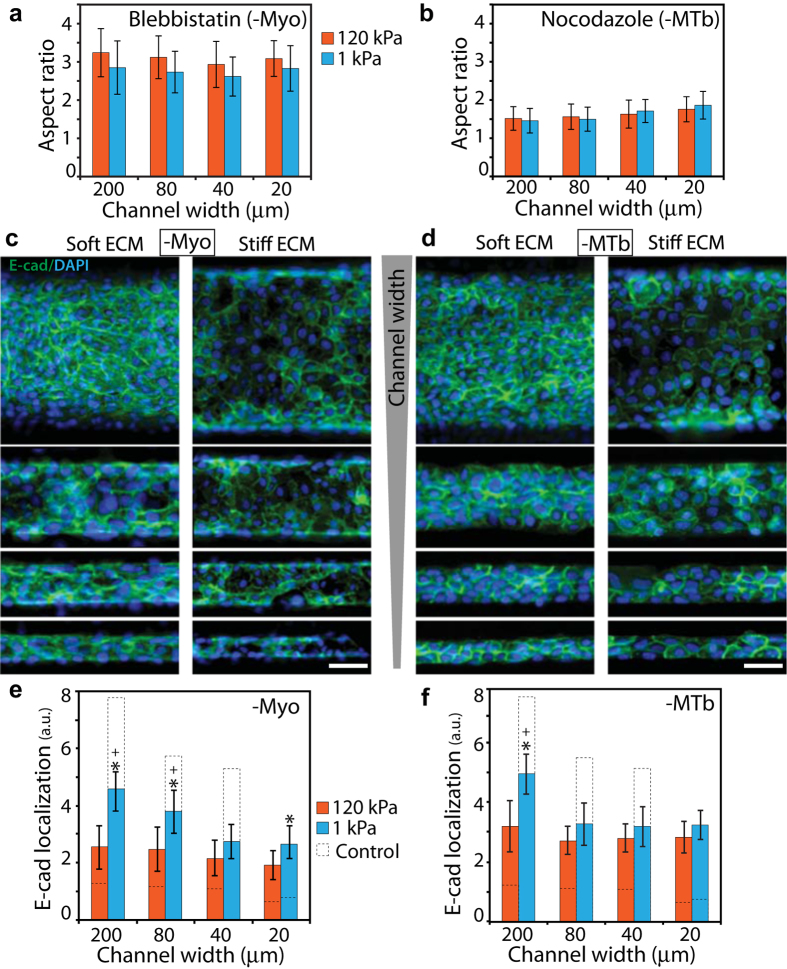
Effects of myosin II and microtubules inhibition on epithelial clusters in confinement. Elongation of cells in the clusters confined inside PA channels, measured as aspect ratio, after **(a)** blebbistatin or **(b)** nocodazole treatment. Representative immunofluorescence images of E-cad (green) expression with DAPI (blue) in **(c)** blebbistatin- and **(d)** nocodazole-treated cells cultured inside channels made of soft and stiff PA gels, with widths between 20–200 μm; see split DAPI and E-cad images in Supplementary Figures 11,12. Scale bar = 50 μm. Average fluorescence intensity of membrane localized E-cad in **(e)** blebbistatin- and **(f)** nocodazole-treated cells cultured in PA channels of varying width and stiffness. ^*^p < 0.05 with respect to stiff ECM. ^+^*p* < 0.05 with respect to narrow (20 μm) channels for 1 kPa stiffness. No significant difference across channel widths for 120 kPa stiffness. N > 8 clusters of numerous cells, from at least three separate experiments (see details in Methods).

To test the connection between elongated cell morphology (Fig. 3c) and enhanced EMT in narrow channels (Figs 2 and 3), we sought to disrupt the ability of cells to rearrange their cytoskeletal structure and elongate in a coordinated manner. We hypothesized that elongation of cells along channel walls might aid their ability to sense ECM confinement. We approached this goal in two different ways. First, we inhibited non-muscle myosin II (Myo) by blebbistatin treatment, which is known to abrogate the generation of active actomyosin forces in cells. While Myo-inhibited cells were more elongated compared to the control cells, they showed no significant differences across channel widths (Fig. 4a). Thus, Myo-inhibition rendered the cells unable to elongate differently in channels of varying confinement. Second, through nocodazole treatment, we inhibited microtubules (MTb) that support the cytoskeletal structure, help stabilize cellular protrusions^25^,^26^, and allow the cells to attain elongated shapes. We found that the elongation of MTb-inhibited cells declined dramatically across all ECM conditions (Fig. 4b). Importantly, even in the narrowest channels (20 μm width) MTb-inhibited cells were unable to exploit confinement and elongate along channel walls.

To examine the respective effects of Myo and MTb inhibition on confinement-sensitive EMT, we imaged E-cad and vimentin distributions for each condition. In stiff ECM, membrane localization of E-cad in Myo-inhibited cells did not vary with channel width (Fig. 4c,e), indicating a confinement-independent EMT. However, in soft ECM, E-cad localization in narrower channels (20 and 40 μm) was lower than that in wider channels (80 and 200 μm), which is similar to the confinement-sensitive trend in control conditions (Fig. 3a). While Myo-inhibition disabled confinement-sensitive EMT in stiff ECMs, it did not have the same effect in soft ECMs (Fig. 4c,e).

**Figure 5:**
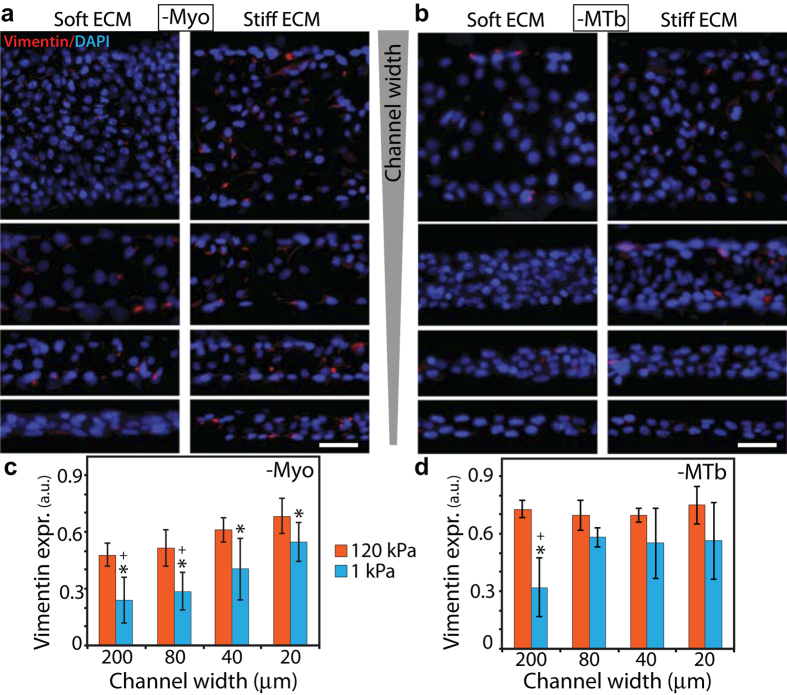
Effects of Myo- and MTb-inhibition on vimentin expression in channel-confined epithelial clusters. Representative immunofluorescence images of vimentin (red) expression with DAPI (blue) in **(a)** blebbistatin- and **(b)** nocodazole-treated cells cultured inside channels made of soft and stiff PA gels, with widths between 20–200 μm; see split DAPI and vimentin images in Supplementary Figures 13,14. Scale bar = 50 μm. Average fluorescence intensity of vimentin in **(c)** blebbistatin- and **(d)** nocodazole-treated cells cultured in PA channels of varying width and stiffness. ^*^p < 0.05 with respect to stiff ECM. ^+^*p* < 0.05 with respect to narrow (20 μm) channels for 1 kPa stiffness. No significant difference across channel widths for 120 kPa stiffness. N > 8 clusters of numerous cells, from at least three separate experiments (see details in Methods).

Next, to assess the role of passive structural properties of the cytoskeleton and MTb-based protrusions in regulating confinement-sensitive EMT, we repeated E-cad measurements after nocodazole treatment. In stiff channels, E-cad expression became insensitive to confinement (Fig. 4d,f). In soft channels, MTb-inhibition was far more effective than Myo-inhibition in reducing the effect of confinement on E-cad localization. Notably, since E-cad expression in soft channels of 20, 40, and 80 μm widths was not significantly different (Fig. 4f), these results indicated that microtubules are essential for epithelial clusters to sense ECM confinement. In other words, after MTb-inhibition, cells in epithelial clusters neither elongated in narrow channels (Fig. 4b), nor underwent confinement-sensitive EMT (Fig. 4d,f). We confirmed these findings of stiffness and confinement sensitive EMT after Myo- or MTb-inhibition by measuring vimentin expression under the same ECM conditions (Fig. 5).

### Inhibition of cell-ECM adhesions reduces the effect of ECM stiffness on EMT

**Figure 6:**
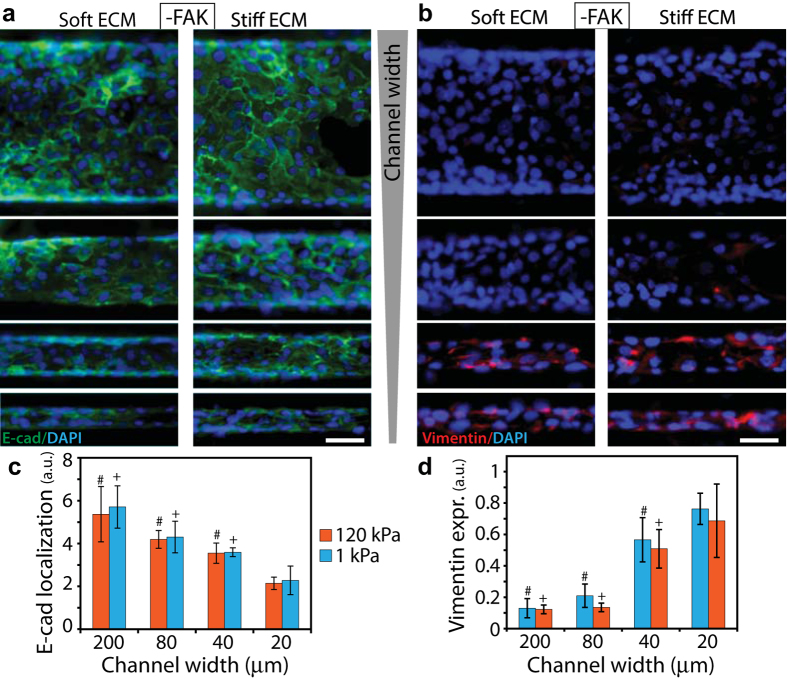
Effect of FAK inhibition on ECM-dependent EMT. Representative immunofluorescence images of **(a)** E-cad (green) and **(b)** vimentin (red) expressions with DAPI (blue) in cells treated with FAK inhibitor PF228 in channels made of soft and stiff PA gels, with widths between 20–200 μm; see split DAPI, E-cad, and vimentin images in Supplementary Figures 15,16. Scale bar = 50 μm. Average fluorescence intensity of **(c)** membrane localized E-cad and **(d)** vimentin expression in FAK-inhibited cells cultured in PA channels of varying width and stiffness. ^+^*p* < 0.05 with respect to narrow (20 μm) channels for 1 kPa stiffness. ^#^*p* < 0.05 with respect to narrow channels for 120 kPa stiffness. No significant difference between soft and stiff ECMs for any channel width. N > 8 clusters of numerous cells, from at least three separate experiments (see details in Methods).

While the disruption of cytoskeletal structure blunted confinement-sensitive cell behavior, stiffness-dependent differences in EMT markers persisted, especially in wide channels (Figs 4e,f and 5c,d). Given that cell-ECM adhesions are key mediators of cellular mechanotransduction^27^,^28^, we hypothesized that the inhibition of focal adhesion kinase (FAK) might disable the cell’s ability to sense ECM stiffness and in turn prevent stiffness-sensitive EMT in channels. To test this hypothesis, we measured the expressions of E-cad (Fig. 6a) in PA channels after pharmacological inhibition of FAK (by treating the cells with PF228). We found that E-cad localization was statistically similar in both soft and stiff ECMs in all channel widths (Fig. 6c). The visualization and quantification of vimentin expression confirmed this trend of stiffness-independent EMT (Fig. 6b,d). Thus, inhibition of cell-ECM adhesions rendered epithelial clusters unable to sense ECM stiffness and undergo EMT in stiffer ECMs. Notably, the E-cad and vimentin expressions varied with channel width in both soft and stiff ECMs, which indicates that the confinement-sensitive EMT persisted even after the disruption of cell-ECM adhesions.

### Relative contributions of subcellular and extracellular inputs in mechanosensitive regulation of EMT

**Figure 7:**
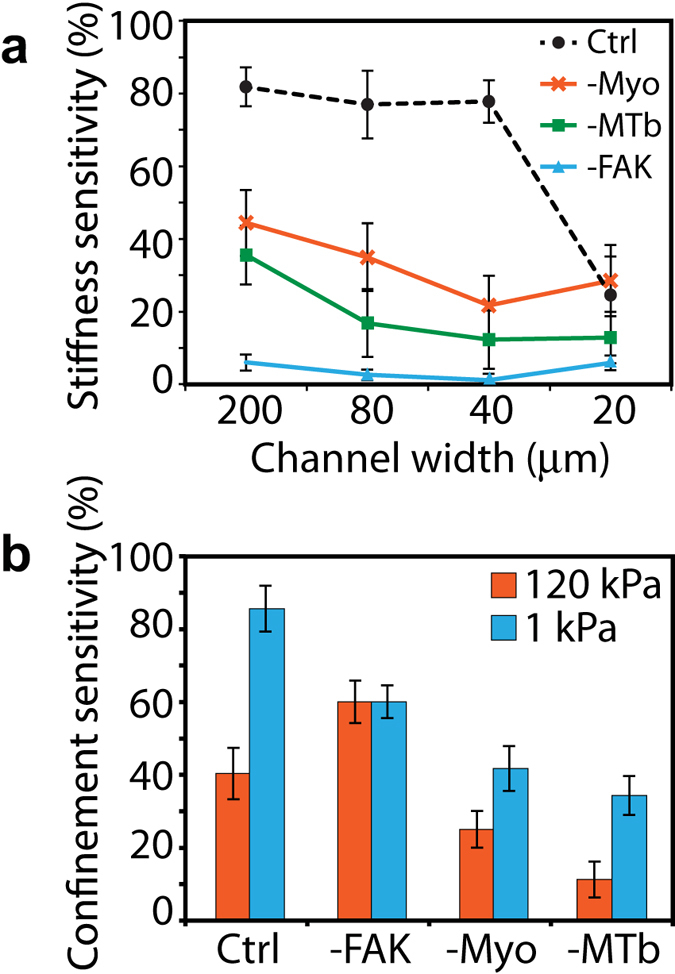
Sensitivity of E-cad localization to extracellular inputs. **(a)** Stiffness sensitivity, calculated as the percentage difference in E-cad localization on stiff and soft ECMs, for any given channel width or knockdown state. **(b)** Confinement sensitivity, calculated as the percentage difference in E-cad localization in wide and narrow channels, for any given stiffness or knockdown state.

To further clarify relative contributions of extracellular inputs to the mechanosensitive regulation of EMT, we calculated “sensitivity” indices corresponding to each ECM property as the percentage change in E-cad localization compared to a reference condition (1 kPa for ‘stiffness sensitivity’ and 200 μm for ‘confinement sensitivity’). First, we calculated ‘stiffness sensitivity’, defined as the percentage change in E-cad localization in stiff ECMs relative to its value in soft ECMs, for any given channel width or knockdown state (Fig. 7a). We found that E-cad localization was most sensitive to stiffness in wide channels across all knockdown (control, –Myo, –MTb, and –FAK) conditions. Interpreted another way, the ability of epithelial clusters to undergo stiffness-dependent EMT went down in confined ECM settings. As noted earlier and confirmed here, the stiffness-sensitivity was highest for control cells and lowest for FAK-knockdown across all channel widths. Next, we computed ‘confinement sensitivity’, defined as the percentage change in E-cad localization in narrow (20 μm) channels relative to its value in wide (200 μm) channels, for any given PA stiffness or knockdown state (Fig. 7b). In both soft and stiff ECMs, control cells were the most confinement sensitive, followed by FAK- and Myo-knockdown states, with MTb-knockdown cells as the least confinement-sensitive. We also noted that epithelial clusters were more confinement sensitive in softer ECM for all knockdown states.

## Discussion

Epithelial clusters of cells emanate from a tumor and interact with the surrounding microenvironment of varying elasticity and geometry. During this interaction with mechanically heterogeneous ECMs, tumor cells in epithelial clusters attain an invasive phenotype by undergoing EMT. While recent studies on different cell types and ECMs have shown that stiffening of the microenvironment can enhance EMT^12^,^15^,^16^,^17^, the influence of matrix topography and confinement on EMT has remained largely unexplored. This gap in knowledge exists mainly due to a marked absence of cell culture platforms that allow independent control over stiffness and confinement of the ECM surrounding the epithelial clusters. In this work, we addressed this challenge by designing a matrix platform for culturing cell clusters in PA channels of varying width and stiffness. Using this device, we have uncovered a previously unidentified confinement-dependent EMT phenotype in soft ECM. While previous studies have indicated that the mechanosensitive EMT remains inactive on soft 2D substrates^15^,^16^, our findings reveal that confined topography of the environment alone can induce EMT even on soft ECMs. On stiffer ECM (120 kPa), mesenchymal transition accelerates regardless of channel width, consistent with previous studies^12^,^15^,^16^,^17^. While it is known that cancer cell clusters of different shapes and sizes can invade tissue microenvironments of different stiffness and topography^18^,^19^, our current understanding of EMT is derived mainly from experiments of epithelial colonies on flat surfaces. Our experimental framework of epithelial clusters in ECMs of defined stiffness and topography can potentially serve as a new platform for studying how groups of cells respond to ECMs that more closely mimic the tissue microenvironments *in vivo*. Through the presented work, we have initiated this effort by identifying that morphological adaptation of cells to the topography of their surrounding environment and the communication of intracellular forces through cell-ECM adhesions are likely central mechanisms by which ECM properties influence the state of cell-cell junctions in epithelial clusters.

Our findings provide a fresh perspective to the emerging body of work on the regulation of mechanosensitive EMT by the mechanical properties of the ECM^12^,^16^,^17^,^29^,^30^,^31^,^32^. In particular, on stiff ECMs, increased cell contractility results in mechano-activation of TGF-β, which in turn leads to dissociation of cell-cell junctions and a down regulation of epithelial markers. It has also been shown that epithelial clusters seeded on topographically aligned fibers experience a elongation of cell shape along the fibers, which leads to increased cell tension, upregulation of TGF-β, and the associated mesenchymal transition^29^,^33^. However, it has been unclear whether the macro-scale confinement of clusters can induce mechanosensitive EMT similar to the phenotypes noted above. Our results fill this important gap in knowledge by revealing that ECM confinement alone is able to induce EMT in epithelial clusters seeded inside narrow channels. Our measurements of E-cad localization over time demonstrate that the mechano-regulation of EMT, either by ECM stiffness or by confinement, benefits from longer duration of culture in stiffer or more confined ECMs. We hypothesized that the ability of epithelial cells to adapt to confined environments by elongating their shape might play an important role in the regulation of confinement-sensitive mesenchymal transition. To test this hypothesis, we targeted microtubules because of their ability to extend protrusions and simultaneously provide compressive mechanical properties to the cytoskeleton during cellular shape changes^34^. Pharmacological inhibition of microtubules abrogated the ability of cells to elongate in confined channels, which led to statistically similar EMT markers across channel width. These results indicate that cells sense and respond to confined environments through adaptive morphological polarization, leading to force alignment in the cell and weakening of cell-cell junctions. However, cell elongation is not the necessary and sufficient condition for creating a friendly environment for EMT induction. The elongated cells also require a stable intracellular force generation machinery to exploit the concentration of forces on cell-cell junctions. We tested this hypothesis by blebbistatin-treatment, which is known to abrogate actomyosin forces and increase cell elongation^35^,^36^. Here, despite increased elongation, blebbistatin-treated cells in confined channels were unable to exert forces required to break cell-cell junctions and induce EMT. These results support the framework in which cells in epithelial clusters require both the actomyosin forces and the ability to elongate in confined ECMs; and, disruption of either one of these mechanisms blunts confinement-sensitive EMT. Surprisingly, cells continued to display different degrees of EMT on soft and stiff ECMs even in the absence of actomyosin forces and the ability to sense confinement. Since the communication of mechanotransductive signaling between the cell and the ECM occurs through focal adhesions, we performed FAK-inhibition and found that epithelial clusters in soft and stiff channels exhibited similar EMT markers. Thus, the stiffness-sensitive EMT requires stable cell-ECM adhesions, which in turn allow actomyosin forces to rise and weaken cell-cell junctions. While actomyosin contractility and focal adhesions are known to cooperatively regulate stiffness sensing in cells^37^,^38^, respective effects of myosin-inhibition on cytoskeletal structure and FAK-inhibition on adhesions alone might lead to different kinds of disruption of stiffness-sensing. Thus, despite their common link of abrogated stiffness sensing, the disrupted cytoskeletal structure of -Myo cells is expected to have different effect on EMT induction than the –FAK cells. In the proposed paradigm of ECM-dependent EMT, while ECM stiffness regulates mechanotransductive signaling and force generation within cells, ECM confinement alters the spatial organization of forces through morphological polarization of cells. Overall, these findings highlight that topographical structure and mechanical stiffness of the tissue microenvironment can both independently regulate EMT, which challenges the current view of matrix stiffness-dependent dissemination of cells from tumor sites.

## Methods

### Fabrication of Polyacrylamide (PA) Device

Silicon master of defined topographical feature was fabricated using a standard photolithography technique. A silicon wafer was cleaned ultrasonically, a layer of SU-8 2015 photoresist (MicroChem) was spin-coated to a thickness of 30 μm, and exposed to 365 nm UV light through a photo-mask of defined geometry. The patterns on the mask are in the form of a well (5 mm diameter), surrounded by channels of widths between 20–200 μm. After dissolving the undeveloped photoresist from the wafer using a SU-8 developer solution (MicroChem), uniformity in channel depth was verified by optical profilometry. Next, the wafer was immersed in Sigmacoat (Sigma Aldrich) for 1 h, washed thoroughly with deionized (DI) water, and dried on the hot plate for 30 mins, which yielded a hydrophobic silicon master. A PA solution, enough to achieve a 100 μm thick gel, was sandwiched between the microchannels master and a reactive coverslip, treated according to an established protocol^4^,^20^. The PA precursor solutions were mixed by choosing monomer:crosslinker ratios based on previous stiffness characterizations of PA gels – acrylamide:bisacrylamide (A:B) percentages of 5%A:0.2%B, 8%A:0.6%B, and 15%A:1.2%B corresponding to PA elastic moduli of 1, 20, and 120 kPa^4^,^21^. Following polymerization of the PA solution, coverslips containing the polymerized PA gels were carefully peeled off from the wafer, which resulted in molded PA gels relative to the topographic features on the master. These PA devices with wells and channels were immersed in PBS and stored at 4 °C before use. The PA devices were UV sterilized for 1 hr and functionalized with extracellular protein by pipetting 1 ml of 0.1 mg.ml^−1^ type I collagen (rat tail collagen, Santa Cruz Technologies) on to the PA surface and incubated overnight at 4 °C. To measure channel height, fluorescent latex micro-beads (Sigma Aldrich) of 0.5μm diameter were mixed with the PA solution (1:250 ratio) and confocal microscopy was performed after polymerization (Supplementary Fig. 1). Using this method, the average channel height was estimated to be approximately 16 μm with a standard deviation of 2.4 μm, from N  >  14 across at least 5 samples for all four channel widths.

### Cell Culture and Immunofluorescence

MCF-10A mammary epithelial cells were grown, as previously described^39^, in DMEM/F12 (Invitrogen), with 5% (v/v) horse serum (Invitrogen), 20 ng/mL epidermal growth factor (EGF, Miltenyi Biotec Inc), 0.5 mg/mL hydrocortisone (Sigma-Aldrich), 100 ng/mL cholera toxin (Sigma-Aldrich), 10 ug/mL insulin (Sigma-Aldrich), and 1% (v/v) penicillin-streptomycin (Sigma-Aldrich). MDCK cells were cultured in MEM (Gibco), with 10% (v/v) FBS (Sigma-Aldrich), 1.5 g/l TC-grade Sodium bicarbonate (Sigma-Aldrich), 1%(v/v) L-glutamine (200 mM stock) (Sigma-Aldrich), 1%(v/v) non-essential amino acids (10 mM stock) (Gibco), 1% (v/v) sodium pyruvate (Sigma-Aldrich), and 10 mg/l gentamycin (50 mg/ml stock) (Sigma-Aldrich). To create an epithelial cell colony, 15,000 cells were seeded in the central chamber of the PA devices, growth media was added, and these cell-laden devices were incubated at 37 °C and 5% CO_2_ for 5days. On day 3 of the assay period, media was replenished for control conditions. For nonmuscle myosin II inhibition, blebbestatin (Sigma-Aldrich; 10 μM) was added on day 4. For microtubules inhibition, nocodozole (Sigma-Aldrich; 5 μM) was added on day 4. For FAK inhibition, PF573228 (Tocris Bioscience; 5 μM) was added on day 3. All samples were fixed on day 5. For vimentin staining, cells were fixed with 4% paraformaldehyde (Santa Cruz Technologies) in PBS, followed by permeabilization of cell membrane with 0.5% Triton-X 100 (Sigma-Aldrich) and blocking with 1% bovine albumin serum (BSA) (EMD milipore). For E-cad staining, cells were fixed in ice-cold methanol (99.5%, Sigma-Aldrich) and blocked with 1% BSA. The following primary antibodies were used: mouse monoclonal E-cadherin (BD Biosciences; diluted 1:500), rabbit monoclonal vimentin (Cell Signaling; diluted 1:200), and mouse monoclonal β-catenin (Santa Cruz Biotechnology; diluted 1:50). The following secondary antibodies were used for immunofluorescence: Alexa Fluor 488-labeled goat anti-mouse, Alexa Fluor 555-labeled goat anti-mouse, and Alexa Fluor 647-labeled goat anti-rabbit (Invitrogen; diluted 1:500).

### Confocal Microscopy and Image Analysis

Confocal microscopy was conducted using a Zeiss LSM 710 laser-scanning confocal microscope (Carl Zeiss Microscopy), where confocal stacks were acquired at 1μm interval and combined using the Z-projection tool in ImageJ (NIH). All image acquisition parameters including laser power, scan speed, and pixel resolution were kept constant to ensure quantitative image analysis. Using the Z-projected E-cad images, the membrane localized E-cad was computed by first subtracting the average cytosolic expression of E-cad in any given cluster of cells (Supplementary Fig. 2b), which were chosen by drawing a region of interest (ROI) around the clusters embedded inside microchannels of defined width. Here, the cytosolic (non-membrane) E-cad expression was computed by measuring pixel values in small regions within individual cells and averaging over at least 7 cells for each ROI. To quantify membrane localized E-cad, the sum of pixel values in any given ROI was normalized by cell density. To ensure the efficacy of this method for quantifying E-cad membrane localization, a second method was employed where E-cad expression was plotted along a line through two adjoining cells and the ratio between cytoplasmic and junctional E-cad expression was noted as “normalized E-cad junctional localization”, as illustrated in Supplementary Figures 2d,e. These calculations were performed for the control case, in all channels widths of both soft and stiff ECMs, and the trends were compared with the first method (Supplementary Figures 2b,c). Based on a general agreement between the calculations from these two methods (compare Supplementary Figs. 2c and 2e), the first method of average E-cad localization (Supplementary Figures 2b,c) is adopted due to its high throughput nature for all measurements presented in the main manuscript. For vimentin expression quantification, the Z-projected vimentin images were analyzed using a custom-built macro in ImageJ to measure the area occupied by the vimentin-expressing intermediate filaments. First, each image was processed by subtracting the background and converting the image to black and white through the binary settings tool in ImageJ. Subsequently, the expression level of vimentin was computed by totaling the number of pixels with a non-zero value and normalizing by cell density in the ROI. For morphological analysis, the shape analysis tool in ImageJ was employed to measure aspect ratios, defined as the ratio of the major axis/minor axis length.

### Statistical Analysis

Data are expressed as the mean ± standard deviation. Statistical significance between means was determined by two-way ANOVA followed by Tukey-Kramer HSD (honestly significant difference) for pairwise comparisons in MATLAB (Mathworks). Means were calculated from N > 8 clusters of numerous cells, corresponding to images acquired from at least three separate experiments. The size of the cell cluster varied with channel width: n > 10 cells/cluster in a 20 μm channel, n > 25 cells/cluster in a 40 μm channel, n > 45 cells/cluster in a 80 μm channel, and n > 90 cells/cluster in a 200 μm channel. For E-cad sensitivity indices (Fig. 7), defined as the percentage difference in means between conditions *x* and *y*, standard deviation was calculated as 
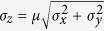
, where 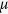 is the ratio of means 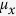 and 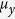, 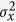 is variance of *x*, and 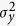 is variance of *y*^40^.

## Additional Information

**How to cite this article**: Nasrollahi, S. and Pathak, A. Topographic confinement of epithelial clusters induces epithelial-to-mesenchymal transition in compliant matrices. *Sci. Rep.* **6**, 18831; doi: 10.1038/srep18831 (2016).

## Supplementary Material

Supplementary Information
