# Supplementary material for: Topographic confinement of epithelial clusters induces epithelial-to-mesenchymal transition in compliant matrices
Source: Sci Rep. 2016 Jan 5;6:18831. doi: 10.1038/srep18831 (PMC4700414; doi:10.1038/srep18831)
Supplement: Supplementary Information [file srep18831-s1.pdf]

SUPPLEMENTARY INFORMATION

for

"Topographic confinement of epithelial clusters induces epithelial-to-mesenchymal transition in compliant matrices"

Samila Nasrollahi and Amit Pathak*

Department of Mechanical Engineering and Materials Science, Washington University, Saint Louis, MO 63130, USA

**Corresponding Author*

Supplementary Figures

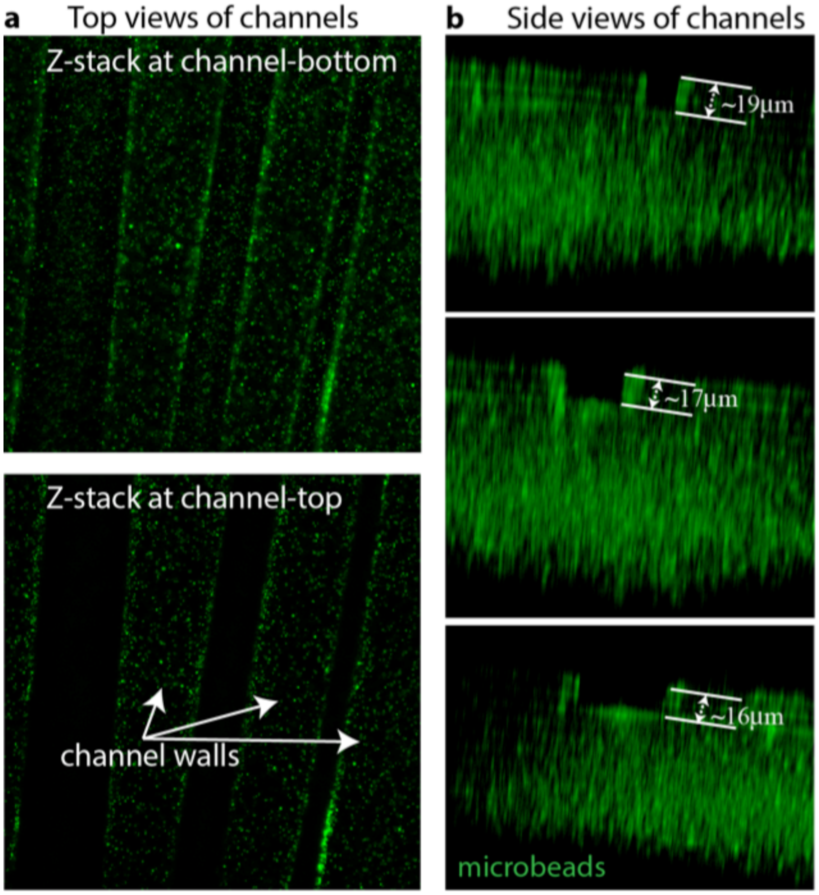

Supplementary Figure 1: Estimation of channel height. (a) Confocal images of fluorescent micro-beads (green) embedded in PA gels showing Z-stacks at the top and the bottom surfaces of the channels. **(b)** Visualizing channel height through lateral views of channels, obtained by 3D volume reconstruction of the confocal Z-stack images of fluorescent micro-beads embedded in PA gels.

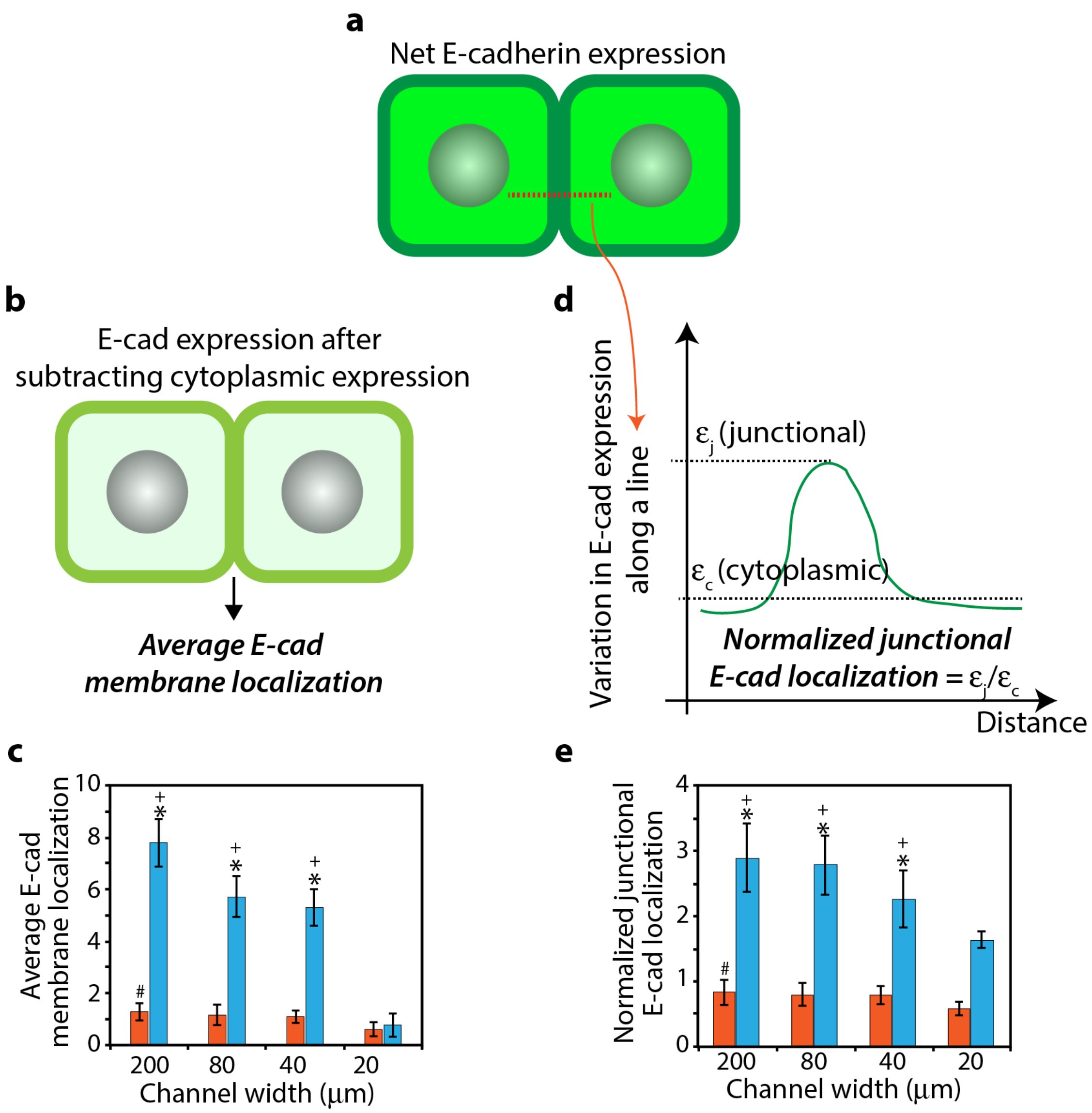

Supplementary Figure 2: Comparison of two methods for quantifying E-cad localization. (a) Schematic describing a typical image of E-cad expression in both the cytoplasm and at intercellular membrane junctions. **(b)** Schematic describing E-cad junctional expression obtained after subtracting the cytoplasmic E-cad expression. **(c)** Average E-cad membrane localization calculated by summing pixel intensity in the subtracted image and normalizing by cell density, for channels of varying width and stiffness. N>8 clusters of numerous cells, from at least three separate experiments (see details in Methods). **(d)** Schematic describing a second method of quantifying E-cad junctional localization in which the E-cad expression along a line drawn between two cells varies such that it reaches a peak value at the intercellular boundary. Here, normalized E-cad junctional localization is computed as the ratio of the junctional E-cad expression, ε_j, and the cytoplasmic E-cad expression, ε_c. **(e)** Normalized E-cad junctional localization ($\varepsilon_j/\varepsilon_c$) calculated by drawing lines between cells inside channels of varying width and stiffness. N>15 pairs of cells per condition, from at least three separate experiments. The general agreement of trends in (c) and (e) demonstrates that the two methods are equivalent. $^*p<0.05$ with respect to stiff ECM. $^+p<0.05$ with respect to narrow (20 μm) channels for 1 kPa stiffness. $^\#p<0.05$ with respect to narrow channels for 120 kPa stiffness.

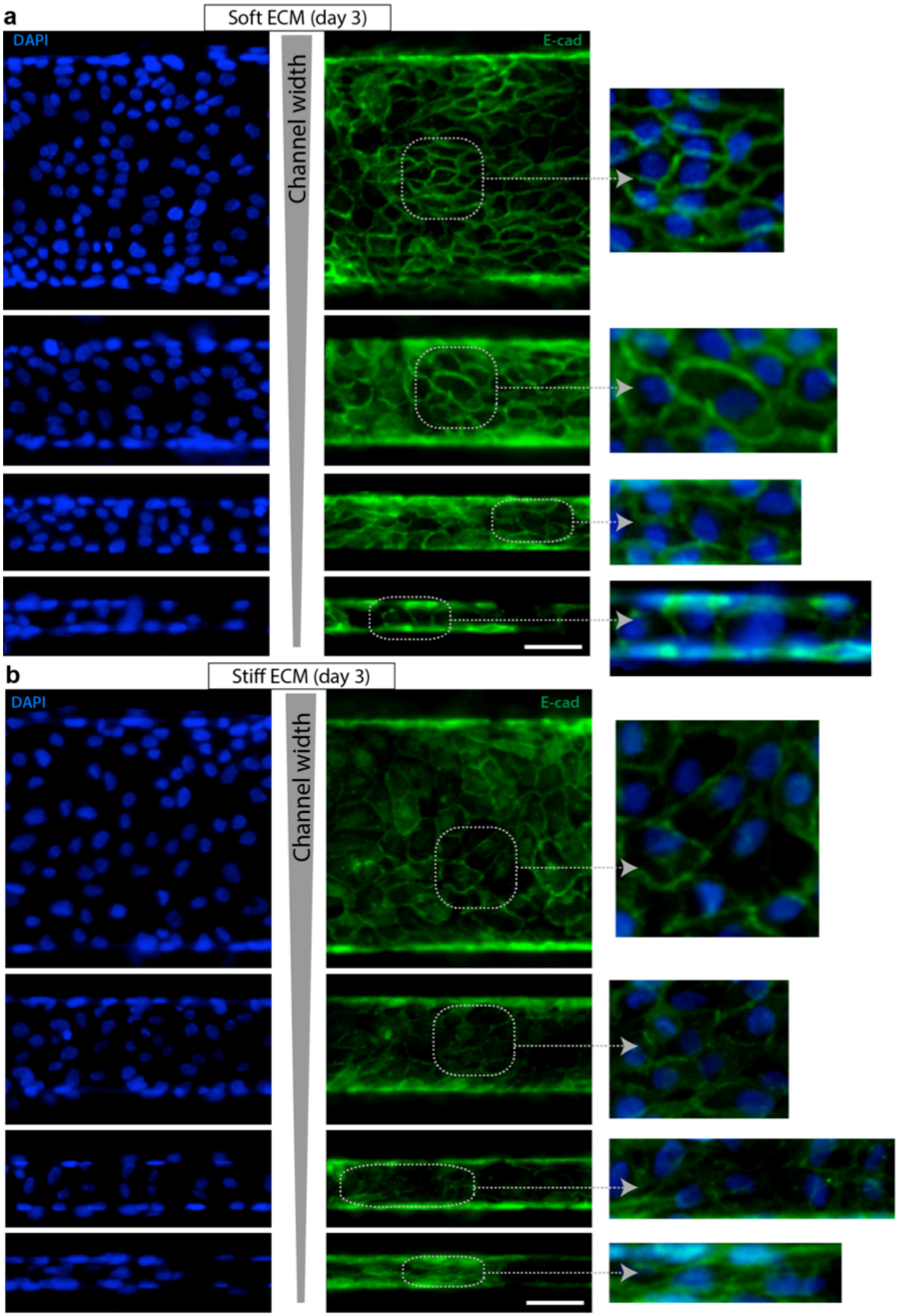

Supplementary Figure 3: Split images of E-cad and DAPI distributions after day 3. Immunofluorescence images of E-cad and DAPI distributions, corresponding to the merged images in Fig. 2, after 3 days of culture inside channels made of **(a)** soft and **(b)** stiff PA gels, with channel widths between 20-200 μm. Scale bar = 50 μm.

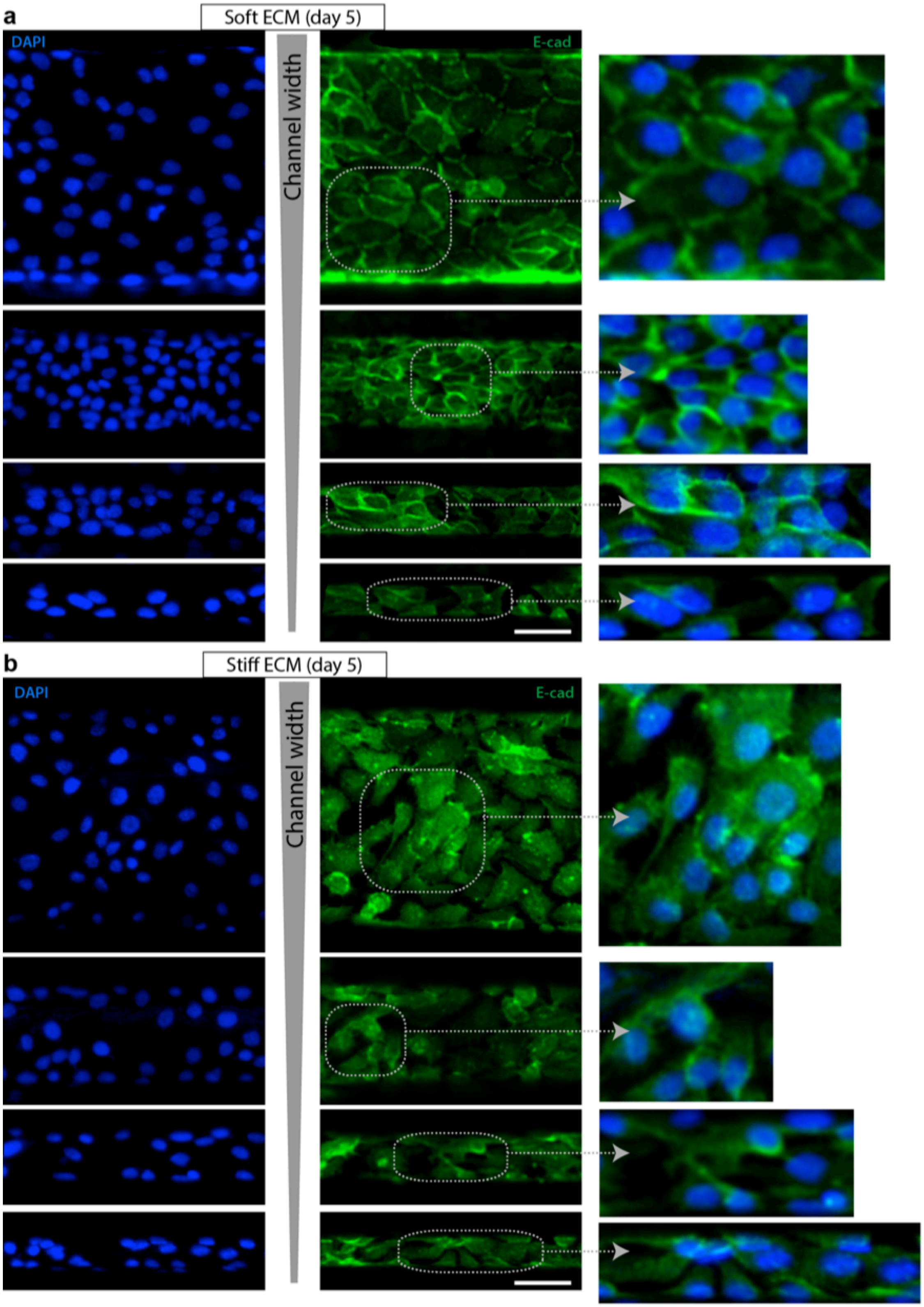

Supplementary Figure 4: Split images of E-cad and DAPI distributions after day 5. Immunofluorescence images of E-cad and DAPI distributions, corresponding to the merged images in Fig. 2, after 5 days of culture inside channels made of **(a)** soft and **(b)** stiff PA gels, with channel widths between 20-200 μm. Scale bar = 50 μm.

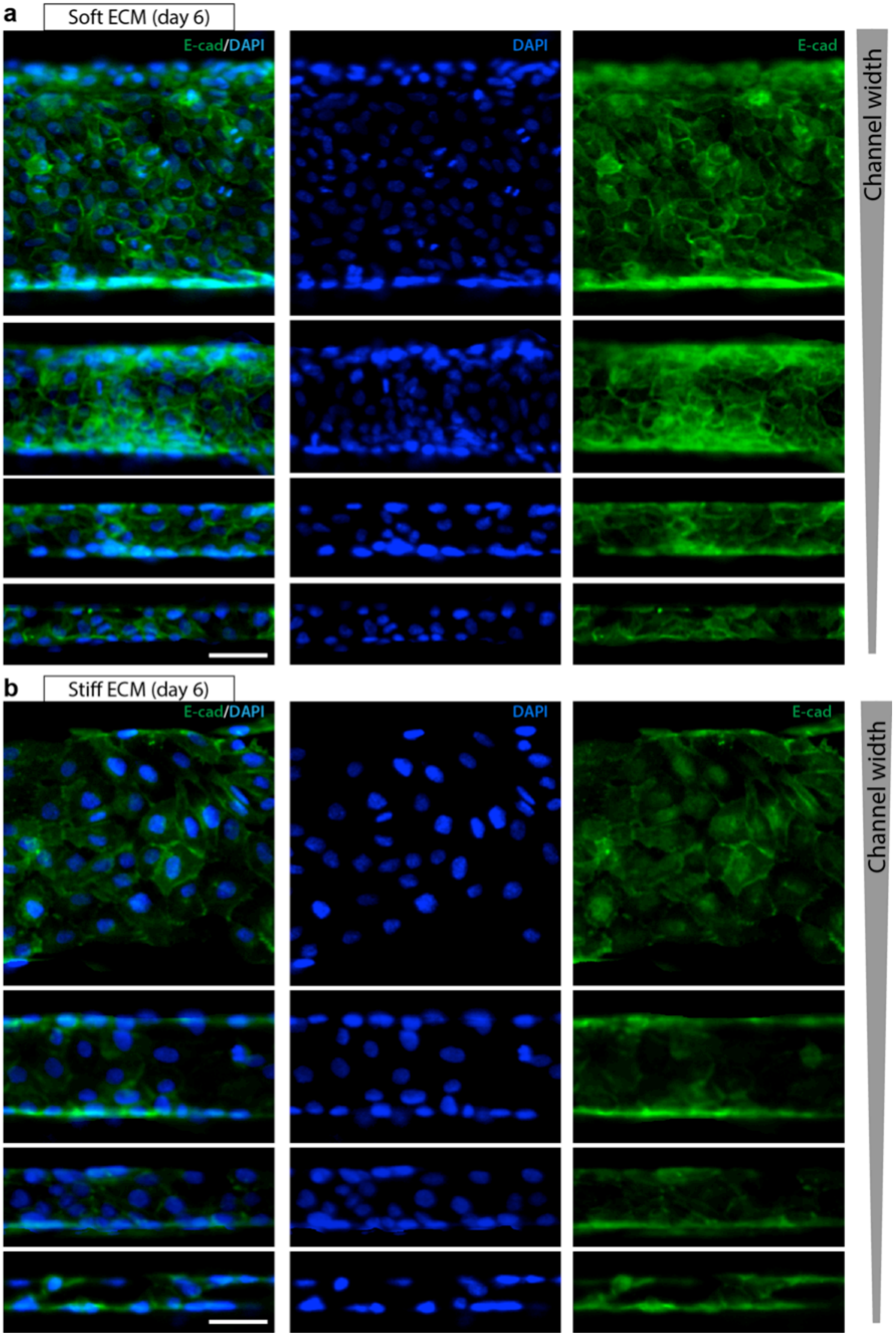

Supplementary Figure 5: Split and merged images of E-cad and DAPI distributions after day 6. Immunofluorescence images of E-cad and DAPI distributions, corresponding to the plots in Fig. 2, after 6 days of culture inside channels made of **(a)** soft and **(b)** stiff PA gels, with channel widths between 20-200 µm. Scale bar = 50 µm.

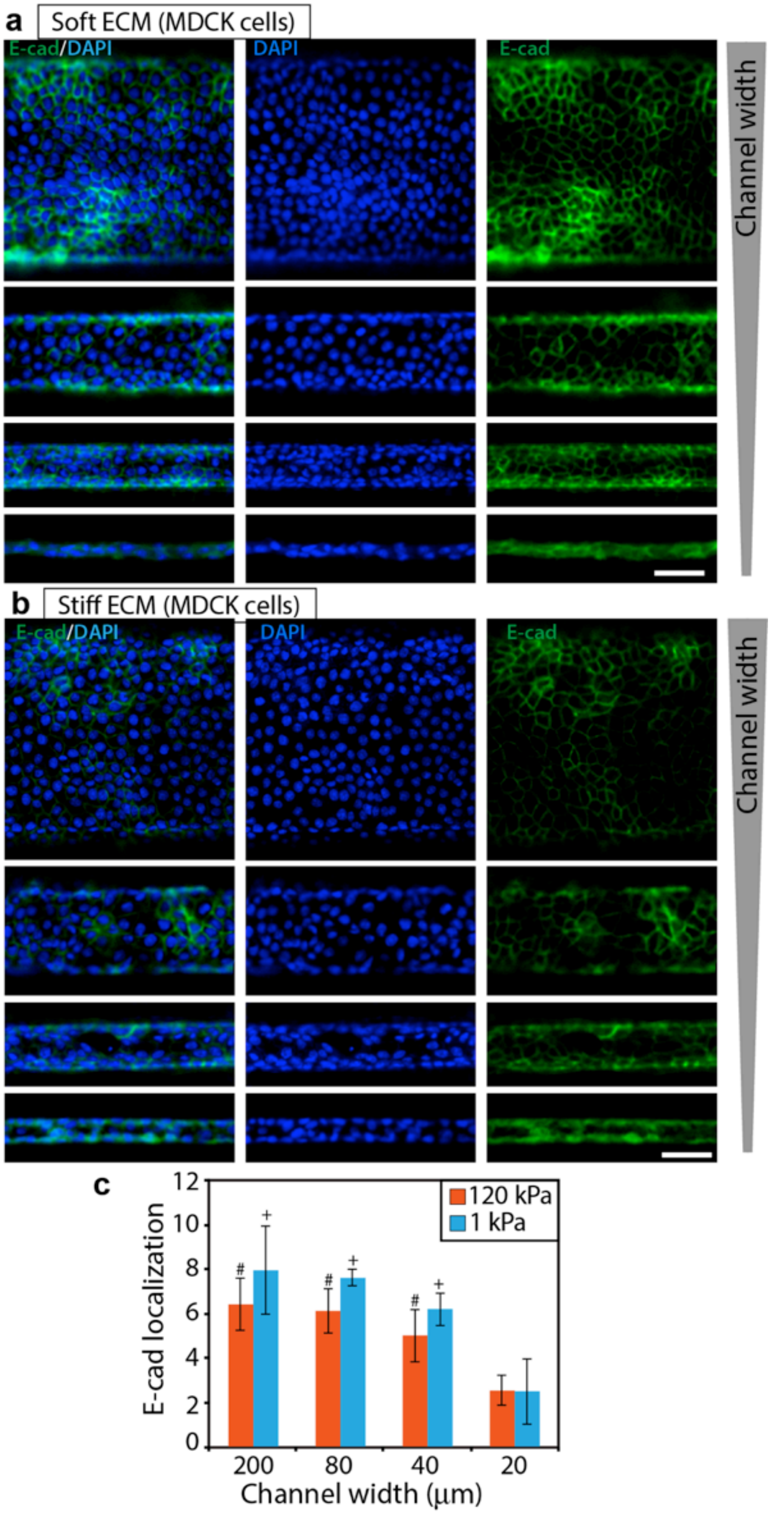

Supplementary Figure 6: Confinement-sensitive EMT in MDCK cells. Representative immunofluorescence images of E-cad (green) expression with DAPI (blue) in MDCK cells cultured inside channels made of **(a)** soft and **(b)** stiff PA gels, with widths between 20-200 µm. Scale bar = 50 µm. **(c)** Average fluorescence intensity of membrane localized E-cad in MDCK cells cultured in PA channels of varying width and stiffness. $^{+}p<0.05$ with respect to narrow (20 µm) channels for 1 kPa stiffness. $^{\#}p<0.05$ with respect to narrow channels for 120 kPa stiffness. No significant difference between soft and stiff ECMs for any channel width. N>6 clusters of numerous cells, from at least three experiments (see details in Methods).

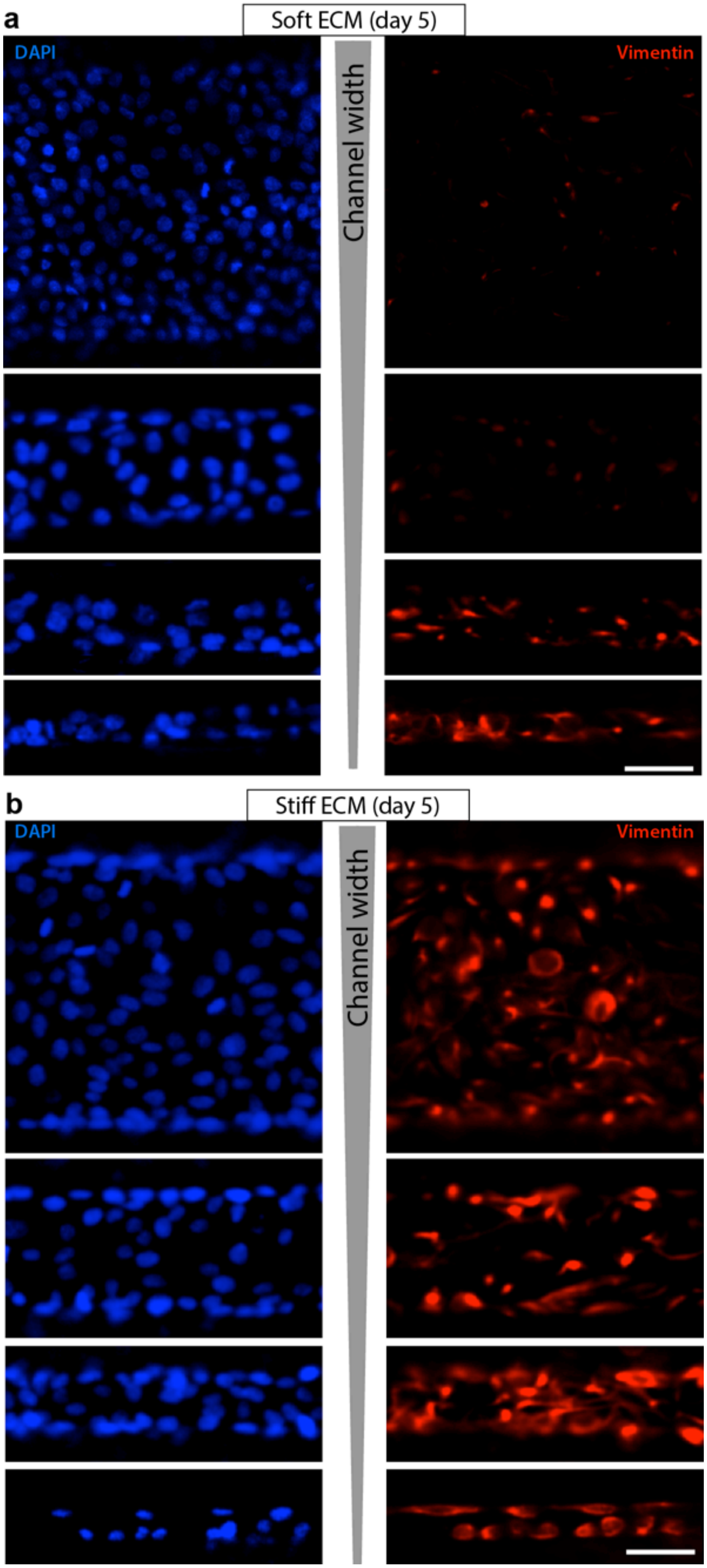

Supplementary Figure 7: Split images of vimentin and DAPI distributions after day 5. Immunofluorescence images of vimentin and DAPI distributions, corresponding to the merged images in Fig. 3, after 5 days of culture inside channels made of **(a)** soft and **(b)** stiff PA gels, with channel widths between 20-200 µm. Scale bar = 50 µm.

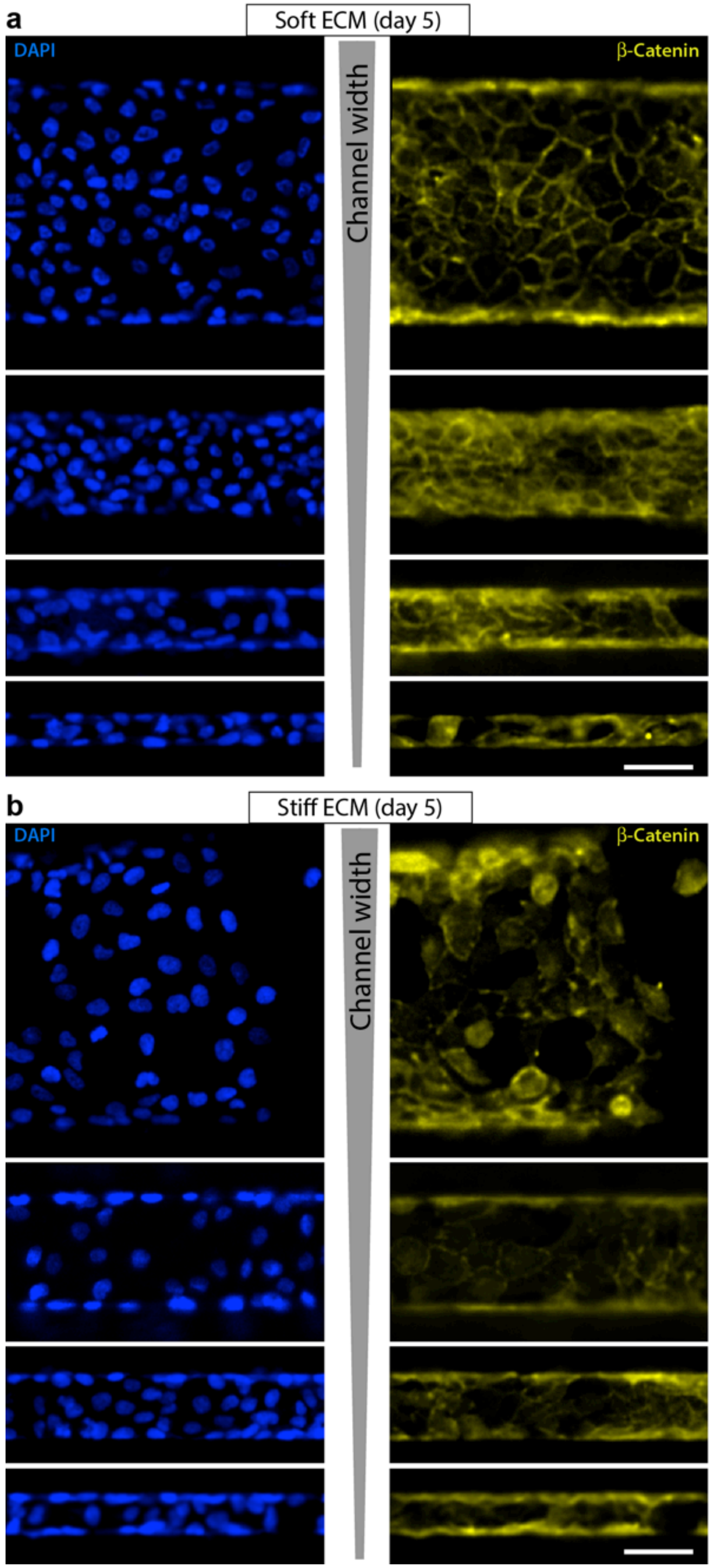

Supplementary Figure 8: Split images of β-catenin and DAPI distributions after day 5. Immunofluorescence images of β-catenin and DAPI distributions, corresponding to the merged images in Fig. 3, after 5 days of culture inside channels made of **(a)** soft and **(b)** stiff PA gels, with channel widths between 20-200 μm. Scale bar = 50 μm.

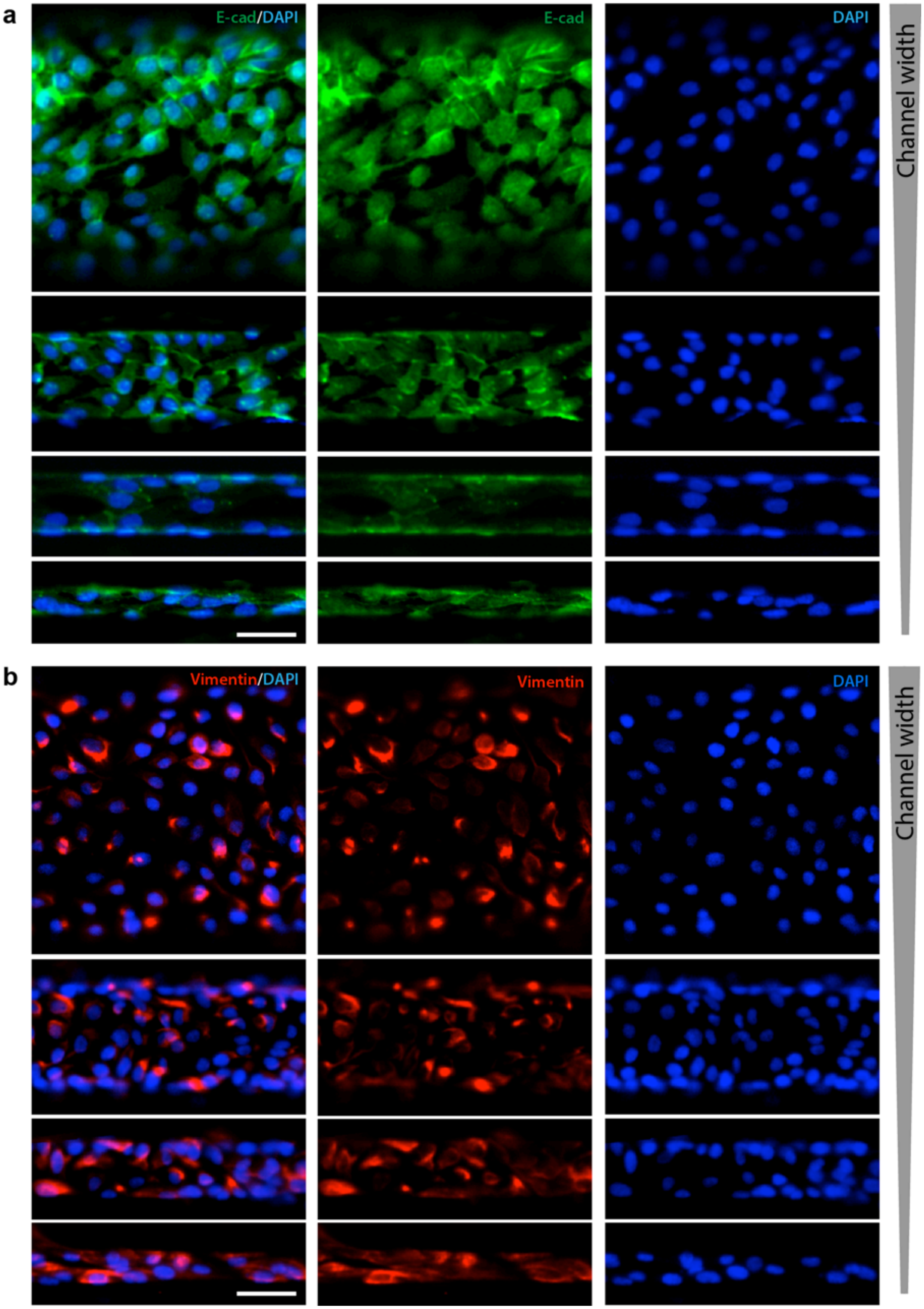

Supplementary Figure 9: E-cad, vimentin and DAPI distributions for ECM stiffness of 20 kPa. Immunofluorescence images of DAPI, **(a)** E-cad, and **(b)** vimentin distributions after 5 days of culture inside channels made of PA gels of 20 kPa stiffness, with channel widths between 20-200 μm. Scale bar = 50 μm.

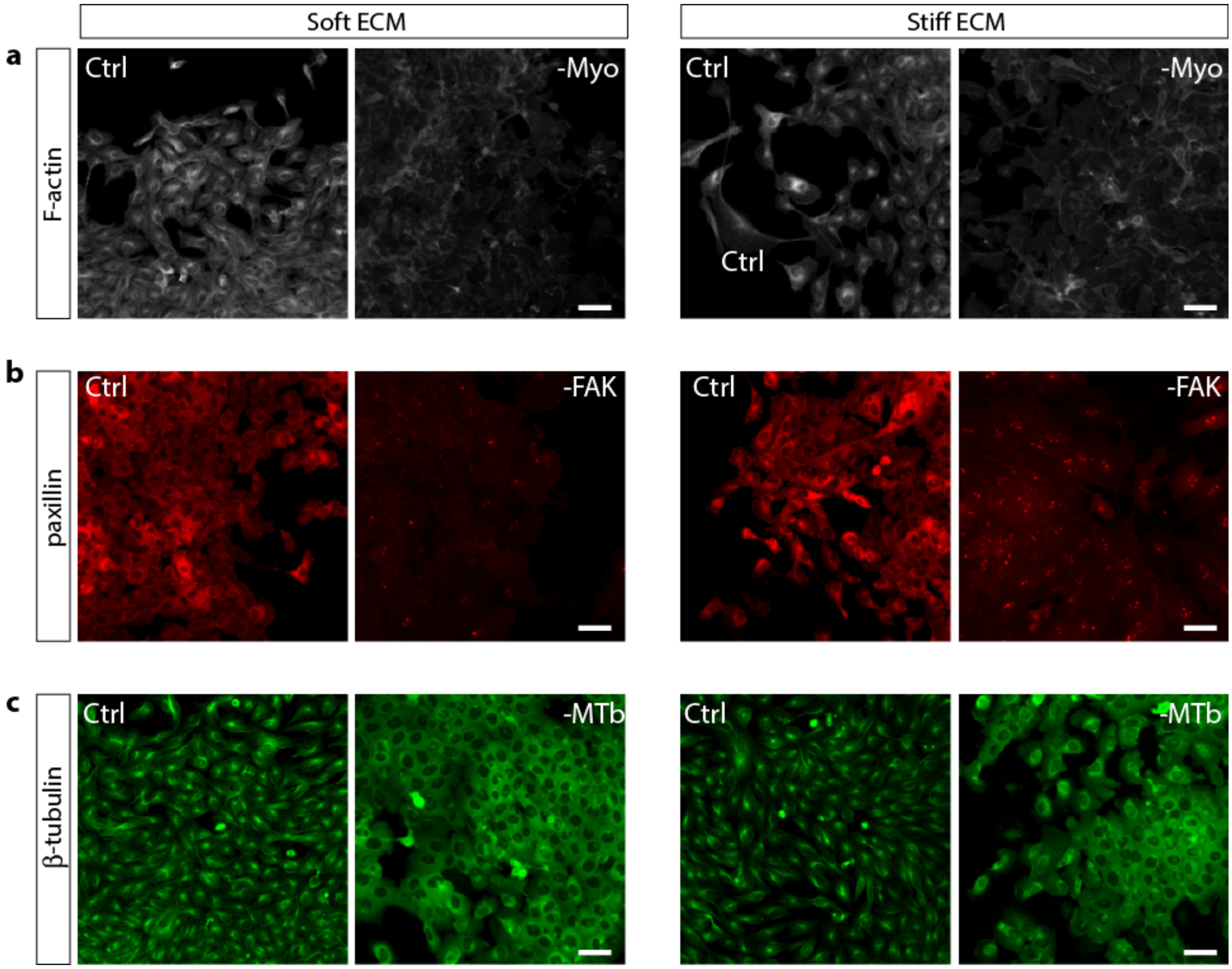

Supplementary Figure 10: Effects of myosin, microtubule, or FAK inhibition. Immunofluorescence images of **(a)** F-actin for control and myosin-inhibited cells, **(b)** paxillin for control and FAK-inhibited cell, and **(c)** β-tubulin for control and microtubule-inhibited cells seeded on soft (1 kPa) and stiff (120 kPa) PA gel substrates. Scale bar = 50 μm.

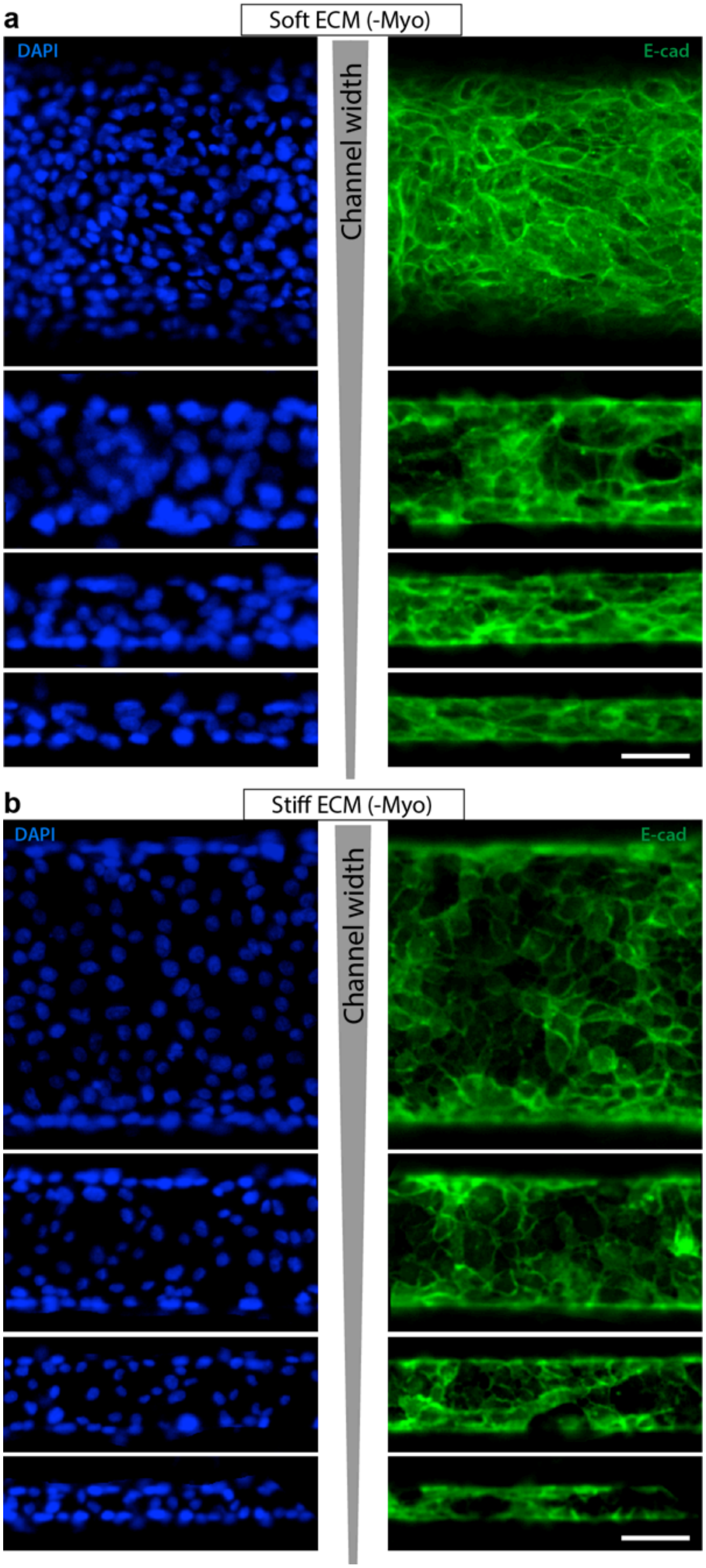

Supplementary Figure 11: Split E-cad and DAPI images after myosin inhibition. Immunofluorescence images of E-cad and DAPI distributions, corresponding to the merged images in Fig. 4, after myosin inhibition inside channels made of **(a)** soft and **(b)** stiff PA gels, with channel widths between 20-200 µm. Scale bar = 50 µm.

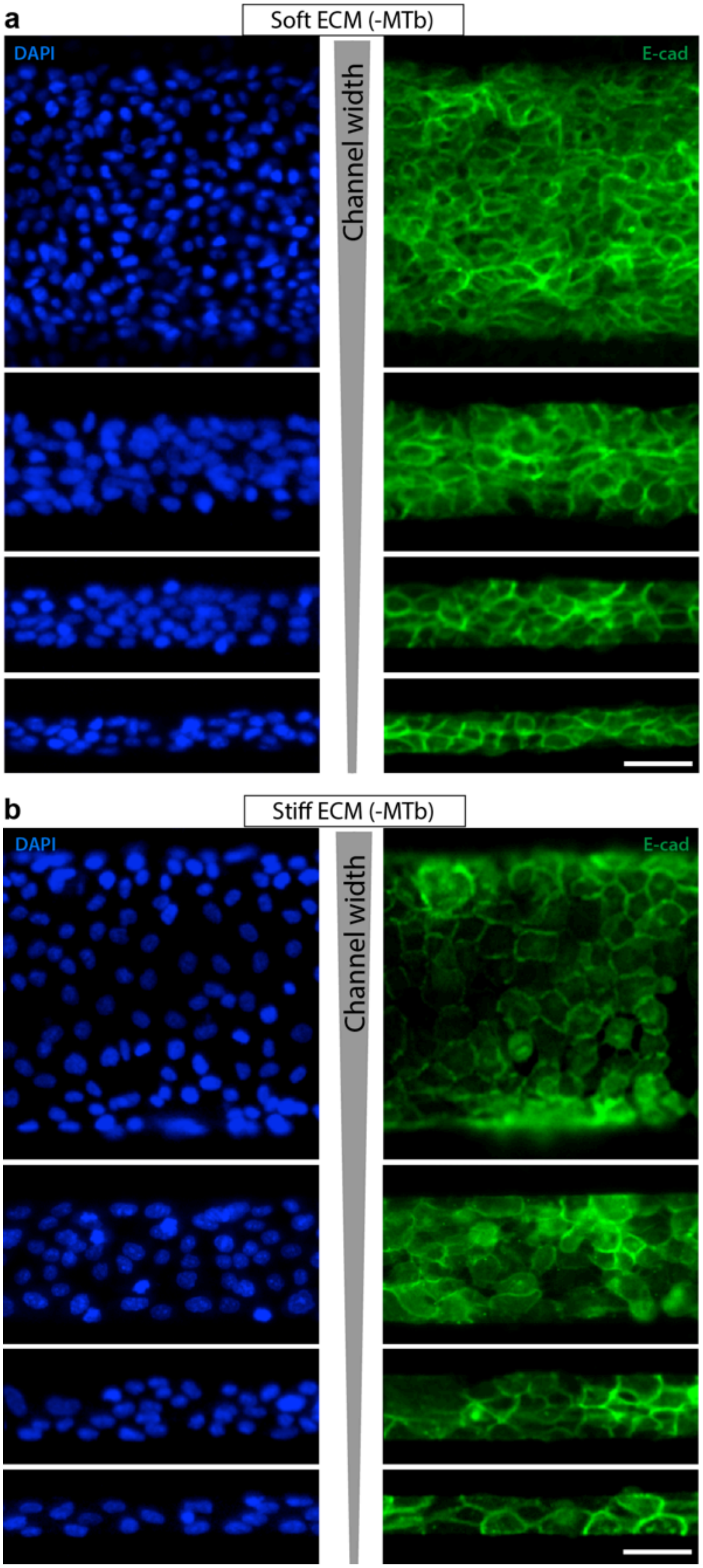

Supplementary Figure 12: Split E-cad and DAPI images after microtubule inhibition. Immunofluorescence images of E-cad and DAPI distributions, corresponding to the merged images in Fig. 4, after microtubule inhibition inside channels made of **(a)** soft and **(b)** stiff PA gels, with channel widths between 20-200 μm. Scale bar = 50 μm.

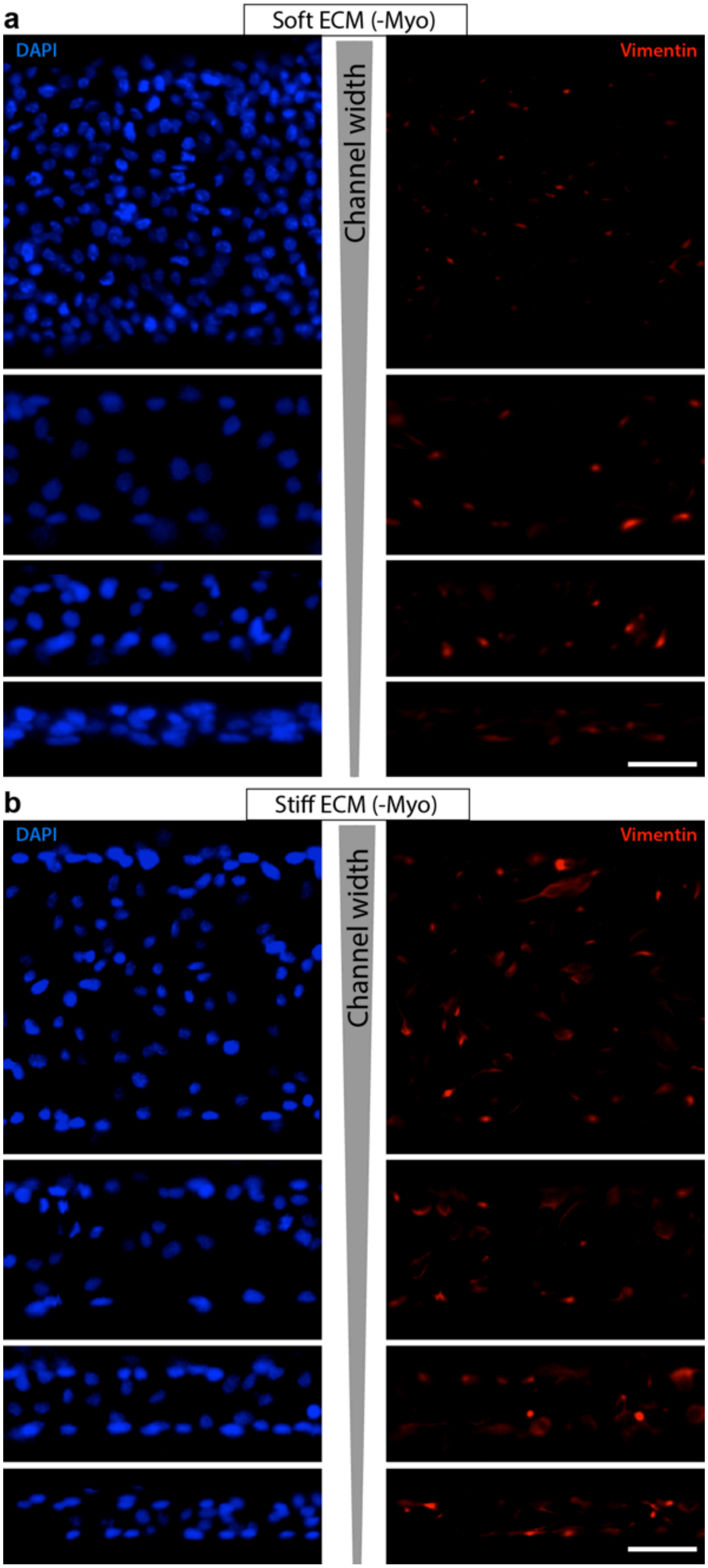

Supplementary Figure 13: Split vimentin and DAPI images after myosin inhibition. Immunofluorescence images of vimentin and DAPI distributions, corresponding to the merged images in Fig. 5, after myosin inhibition inside channels made of **(a)** soft and **(b)** stiff PA gels, with channel widths between 20-200 μm. Scale bar = 50 μm.

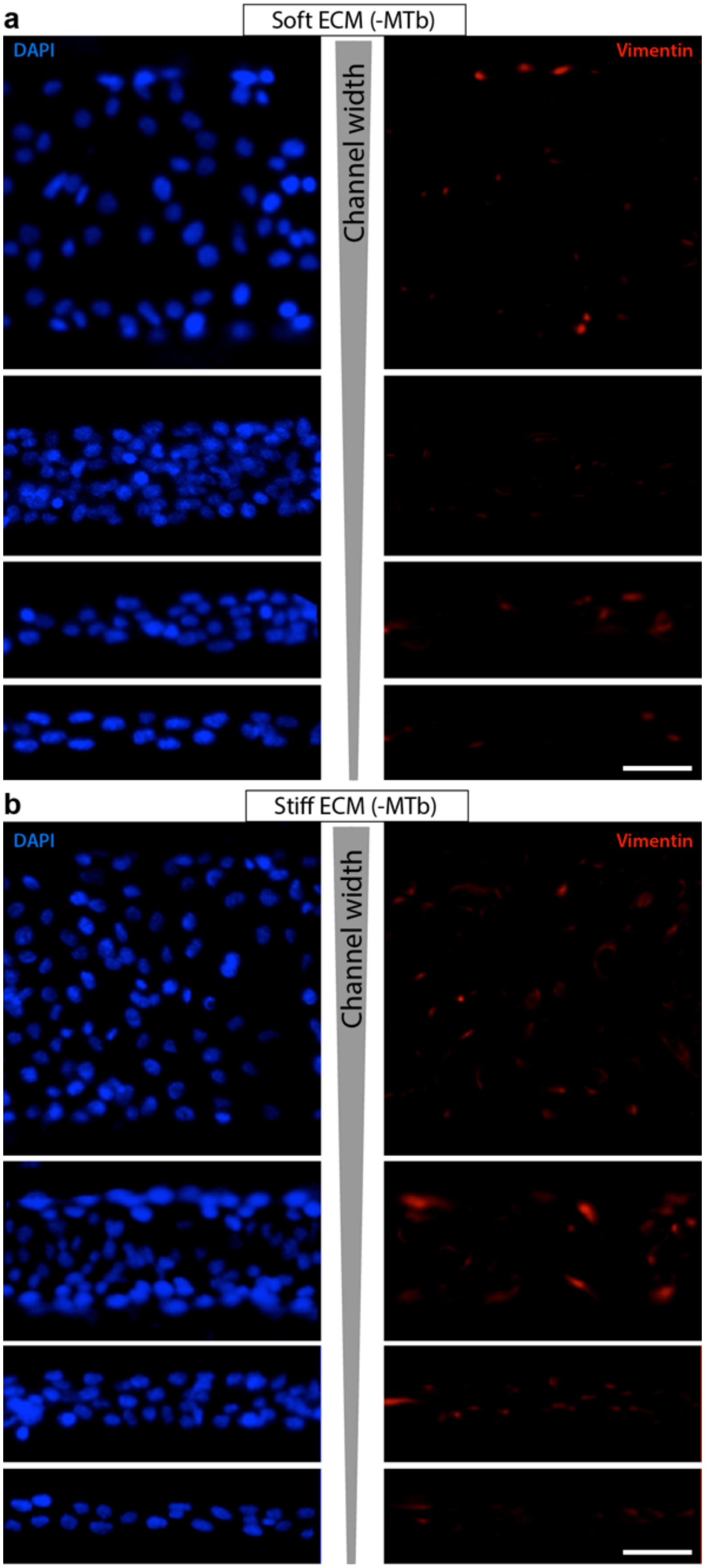

Supplementary Figure 14: Split vimentin and DAPI images after microtubule inhibition. Immunofluorescence images of vimentin and DAPI distributions, corresponding to the merged images in Fig. 5, after microtubule inhibition inside channels made of **(a)** soft and **(b)** stiff PA gels, with channel widths between 20-200 µm. Scale bar = 50 µm.

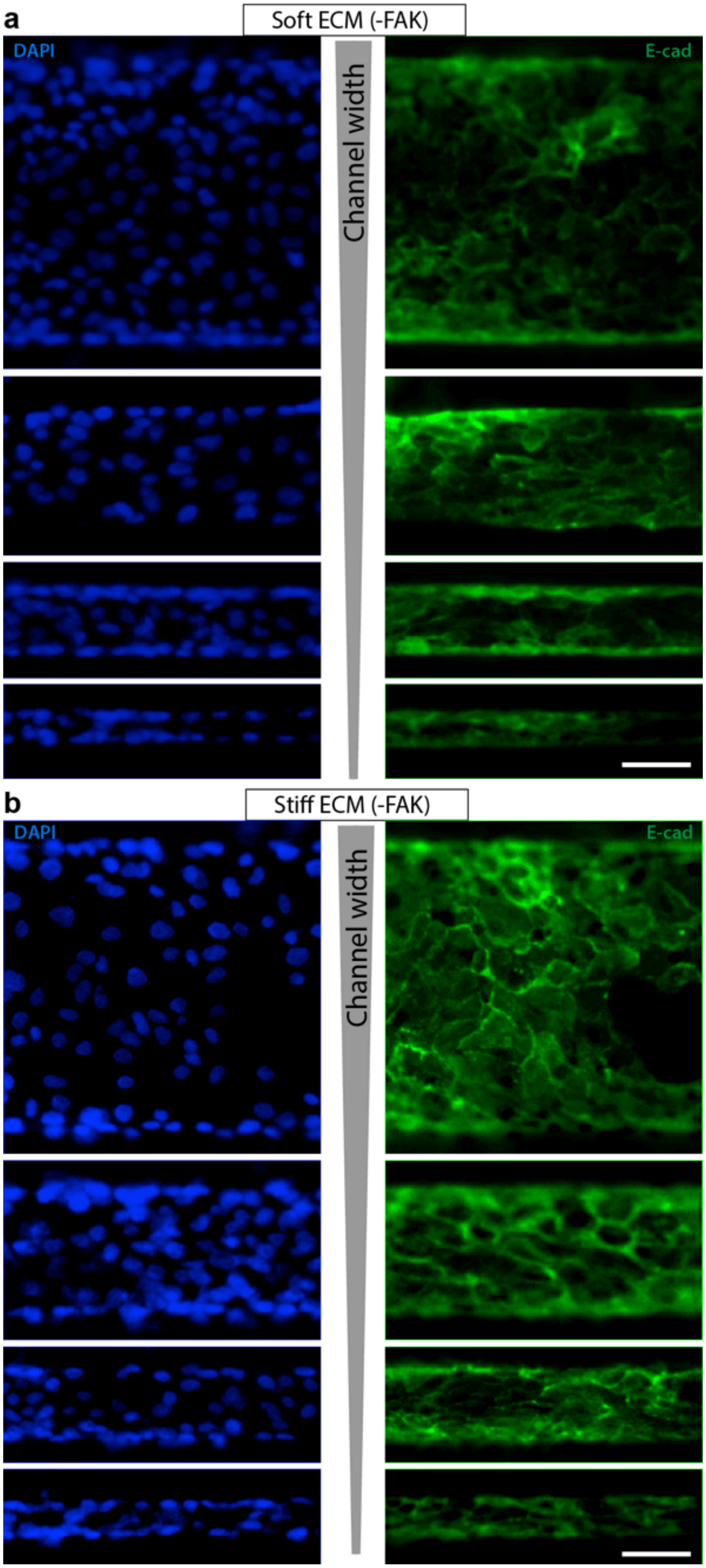

Supplementary Figure 15: Split E-cad and DAPI images after FAK inhibition. Immunofluorescence images of E-cad and DAPI distributions, corresponding to the merged images in Fig. 6, after FAK inhibition inside channels made of **(a)** soft and **(b)** stiff PA gels, with channel widths between 20-200 µm. Scale bar = 50 µm.

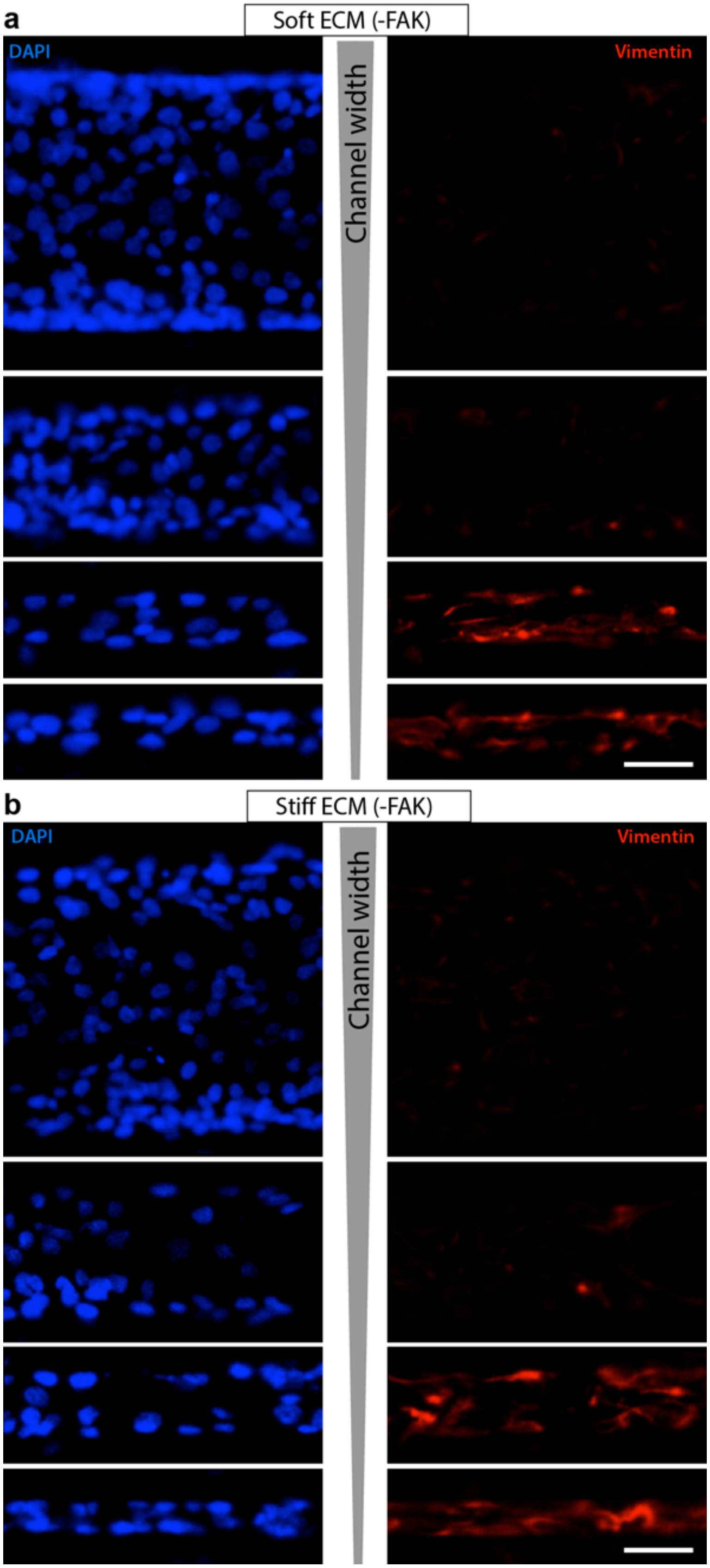

Supplementary Figure 16: Split vimentin and DAPI images after FAK inhibition. Immunofluorescence images of vimentin and DAPI distributions, corresponding to the merged images in Fig. 6, after FAK inhibition inside channels made of **(a)** soft and **(b)** stiff PA gels, with channel widths between 20-200 µm. Scale bar = 50 µm.
